# Analysing a Group of Homologous BAHD Enzymes Provides Insights into the Evolutionary Transition of Rosmarinic Acid Synthases from Hydroxycinnamoyl-CoA:Shikimate/Quinate Hydroxycinnamoyl Transferases

**DOI:** 10.3390/plants13040512

**Published:** 2024-02-13

**Authors:** Jiali Zhou, Xiaofang Zou, Zixin Deng, Lian Duan

**Affiliations:** Key Laboratory of Combinatory Biosynthesis and Drug Discovery, Ministry of Education, School of Pharmaceutical Science, Wuhan University, Wuhan 430071, China; 2021203060022@whu.edu.cn (J.Z.); 2022283060061@whu.edu.cn (X.Z.); zxdeng@sjtu.edu.cn (Z.D.)

**Keywords:** homologous protein, hydroxycinnamoyl-CoA:shikimate/quinate hydroxycinnamoyl transferase, promiscuity, rosmarinic acid synthase, evolution

## Abstract

The interplay of various enzymes and compounds gives rise to the intricate secondary metabolic networks observed today. However, the current understanding of their formation and expansion remains limited. BAHD acyltransferases play important roles in the biosynthesis of numerous significant secondary metabolites. In plants, they are widely distributed and exhibit a diverse range of activities. Among them, rosmarinic acid synthase (RAS) and hydroxycinnamoyl-CoA:shikimate/quinate hydroxycinnamoyl transferase (HCT) have gained significant recognition and have been extensively investigated as prominent members of the BAHD acyltransferase family. Here, we conducted a comprehensive study on a unique group of RAS homologous enzymes in *Mentha longifolia* that display both catalytic activities and molecular features similar to HCT and Lamiaceae RAS. Subsequent phylogenetic and comparative genome analyses revealed their derivation from expansion events within the HCT gene family, indicating their potential as collateral branches along the evolutionary trajectory, leading to Lamiaceae RAS while still retaining certain ancestral vestiges. This discovery provides more detailed insights into the evolution from HCT to RAS. Our collective findings indicate that gene duplication is the driving force behind the observed evolutionary pattern in plant-specialized enzymes, which probably originated from ancestral enzyme promiscuity and were subsequently shaped by principles of biological adaptation.

## 1. Introduction

Numerous plants belonging to the Lamiaceae family have been extensively employed in traditional medicinal practices for the treatment of diverse ailments, including inflammation, infection, depression, weakness, and indigestion [1,2,3]. The results of further investigations have revealed that some of the therapeutic properties of these plants primarily stem from their abundant content of phenolic compounds, with a specific emphasis on rosmarinic acid (RA) [4,5]. Pharmacological studies have demonstrated that RA exhibits a wide range of activities encompassing anti-inflammatory effects [6], antioxidative properties [7], antidiabetic potential [8], antitumor activity [9], antivirus effects [10], neuroprotective actions [11], and hepatoprotective benefits [12]. Meanwhile, within the plant kingdom, RA is commonly recognized as an adaptive mechanism to cope with challenging environments and primarily functions in mediating interspecific interactions from a defensive perspective [13].

**Figure 1:**
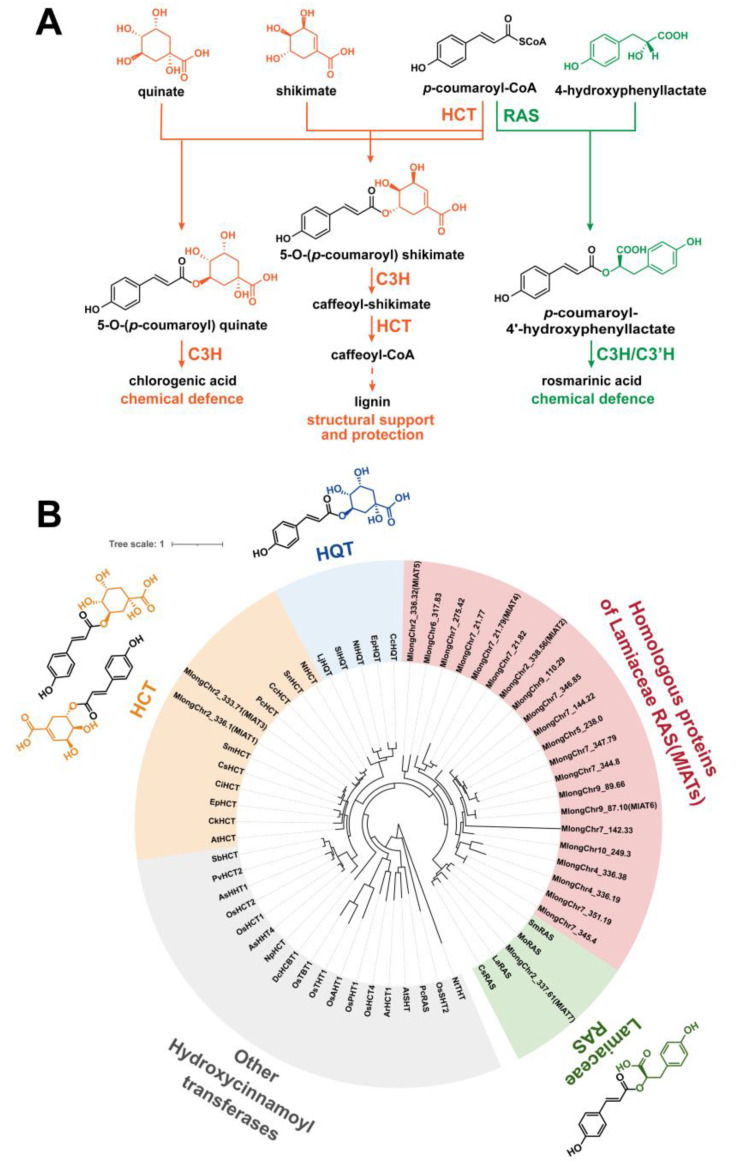
(**A**) In plants, HCT primarily catalyses the transfer of the *p*-coumaroyl acyl group from *p*-coumaroyl-CoA to shikimate or quinate to produce *p*-coumaroyl shikimate or *p*-coumaroyl quinate, while RAS mainly accepts *p*-coumaroyl-CoA and 4-hydroxyphenyllactate to produce *p*-coumaroyl-4′-hydroxyphenyllactate, a main precursor of rosmarinic acid. The final products of these metabolic pathways assume roles in phytochemical defence or structural support. HCT, shikimate/quinate hydroxycinnamoyl transferase; RAS, rosmarinic acid synthase; C3H, *p*-coumaroyl 3-hydroxylase; C3′H, *p*-coumaroyl 3′-hydroxylase. (**B**) The phylogenetic analysis of *Ml*ATs. This phylogenetic tree displays the evolutionary connections between *Ml*ATs and diverse hydroxycinnamoyl transferases originating from distinct plant taxa. *Ml*AT1 and *Ml*AT3 are grouped within the clade of the HCT family. Other *Ml*ATs are adjacent to the Lamiaceae RASs. HQT, quinate hydroxycinnamoyl transferase. *Ml*ATs, a general term for RAS homologous proteins derived from *Mentha longifolia*.

In order to further investigate and exploit the potential of RA, researchers conducted an analysis of the biosynthetic pathway in plants, leading to the discovery of a crucial enzyme known as rosmarinic acid synthase (RAS). RAS facilitates the transfer of *p*-coumaroyl acyl groups from *p*-coumaroyl-CoA to the 4-hydroxyphenyllactate compound, resulting in the production of *p*-coumaroyl-4’-hydroxyphenyllactate, which serves as a precursor for RA (Figure 1A). Several RASs have been isolated from various species belonging to Lamiaceae and Boraginaceae families, renowned for their extensive distribution and high accumulation of RA. Interestingly, these enzymes were believed to have evolved from two distinct pathways. Lamiaceae RASs exhibit significant sequence homology with shikimate/quinate hydroxycinnamoyl transferases (HCTs), a kind of early-evolved enzyme [13,14], while Boraginaceae RASs were thought to originate from a spermidine hydroxycinnamoyl transferase (SHT) ancestor [15]. Notably, SHT was also considered as an enzyme family that has expanded and evolved from HCT [16,17].

As an early-evolved plant-specialized metabolic enzyme, HCTs exhibited functional conservation over 500 million years of embryophyte development, and plays a crucial role in the phenylpropanoid pathway [18]. This pathway generates a vast array of specialized metabolites and precursors of significant extracellular biopolymers that facilitated early adaptation to terrestrial environments [17,19,20]. Members of the HCT family belong to clade V of BAHD acyltransferases [21] and consistently utilize shikimate and quinate as preferred acyl acceptors to form *p*-coumaroyl shikimate esters and *p*-coumaroyl quinate esters (Figure 1A) [22,23,24,25]. Furthermore, investigations into the catalytic properties of HCT have revealed its broad substrate permissiveness towards acceptor molecules [22,26,27,28]. The promiscuous catalytic property may serve as a foundation for the subsequent emergence of hydroxycinnamoyl transferases with novel functionalities.

Previous investigations have suggested that Lamiaceae RAS might share a pedigree with HCT [14]. Notably, these studies also highlighted significant divergence in the substrate binding centres between the two types of enzymes, which accounts for their distinct substrate selectivity [13,14,27]. However, the preliminary discussion of evolution at the sequence level does not provide substantial evidence to determine the specific differentiation relationship between RAS and HCT. Although current hypotheses lean towards Lamiaceae RAS being derived from an ancient HCT ancestor [15,17], further investigations are required to draw definitive conclusions and explore more intricate details. This study focuses on elucidating the evolutionary hypothesis of Lamiaceae RAS by integrating structural, functional, and evolutionary aspects through the incorporation of additional intermediate sequences to identify deeper insights into these hydroxycinnamoyl transferases.

## 2. Results

### 2.1. A Large Number of RAS Homologous Genes Distributed in the Genome of Menta longifolia

The amino acid sequences of RAS enzymes contributing to the formation of RA in Lamiaceae plants exhibit a significant degree of correlation. For instance, *Cs*RAS (from *Coleus scutellarioides*) shares a pairwise sequence identity of 79.5% with *Sm*RAS (from *Salvia miltiorrhiza*), 79.6% with *La*RAS (from *Lavandula angustifolia*), and 82.3% with *Mo*RAS (from *Melissa officinalis*). To gain deeper insights into the origin and development of RAS, these enzymes were employed as baits to identify their homologues in the genome of *Mentha longifolia*, a mint species that exhibits a high level of RA accumulation [29] and has been established as a model species for genetic and genomic research on mint [30]. However, our genome-based exploration did not yield strong related candidate genes. Interestingly, numerous RAS homologous genes are distributed throughout the *Menta longifolia* genome, primarily concentrated on chromosomes 2, 7, and 9. The enzymes encoded by these RAS homologous genes were designated as *Ml*ATs for the sake of simplicity. Preliminary analysis indicates that all *Ml*ATs belong to the plant BAHD acyltransferase family.

To elucidate these RAS homologous genes, we constructed a phylogenetic tree encompassing various types of hydroxycinnamoyl transferases (Figure 1B). The phylogenetic tree demonstrated that most of hydroxycinnamoyl transferases were grouped based on physiological/biochemical functions. HCTs and Lamiaceae RASs were classified into distinct clades. The Boraginaceae *Pc*RAS (from *Phacelia campanularia*) was divided into an individual clade, which was consistent with a previous report of independent evolution [15]. In the *Ml*ATs population, *Ml*AT1 and *Ml*AT3 were located in the clade of HCT, while the majority of other *Ml*ATs were grouped into a separate clade neighbour to the Lamiaceae RAS family. The conservation level of the sequences within this clade is relatively low compared with other types of enzymes on the tree, with certain members displaying only 50% to 60% identity. *Ml*AT7 appears to belong to the Lamiaceae RAS family; however, the enzyme encoded by this gene consists of only 160 amino acids, lacking a complete acyltransferase structure.

### 2.2. Revealing the Primary Activity of MlATs through Comprehensive Characterization

As homologous enzymes of RAS, *Ml*ATs may also be involved in the biosynthesis of RA. Therefore, we selected several enzymes (*Ml*AT1, *Ml*AT2, *Ml*AT4, and *Ml*AT6) for in vitro functional verification. These selected enzymes exhibit a certain degree of evolutionary divergence and originate from distinct chromosomes. For testing purposes, salvianolic acid A, 4-hydroxyphenyllactate, shikimate, and quinate were chosen as prioritized acyl acceptors, while *p*-coumaroyl-CoA and caffeoyl-CoA were chosen as acyl donors in the enzymatic reactions.

**Figure 2:**
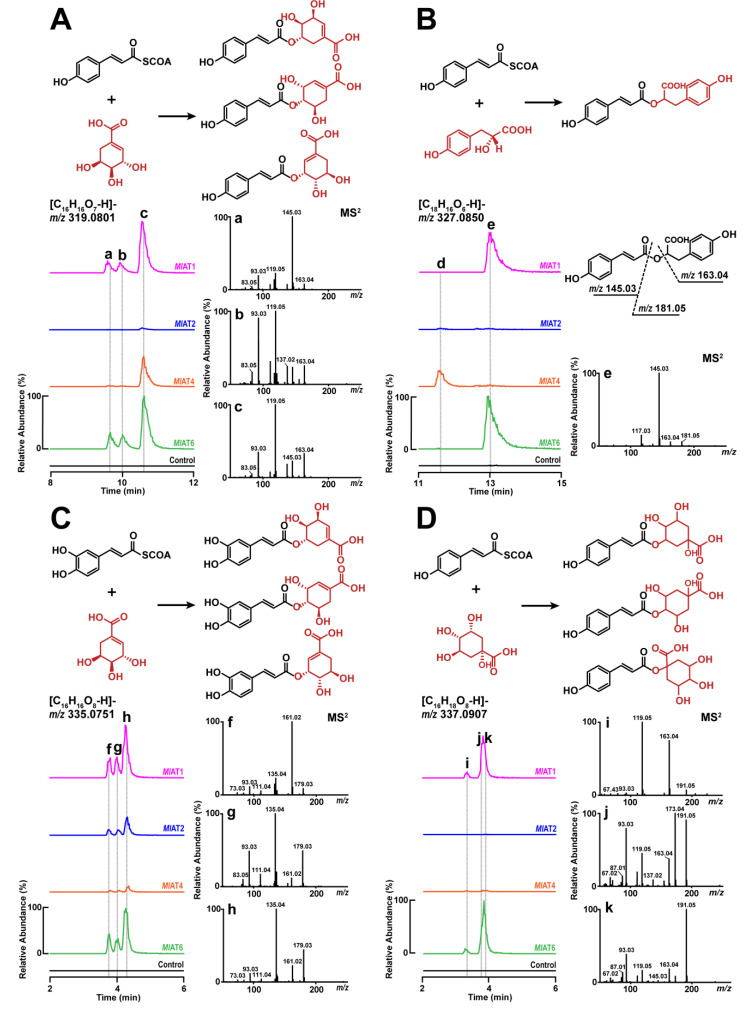
Enzyme activity test of *Ml*ATs. Acyl acceptors, namely 4-hydroxyphenyllactate, shikimate, and quinate, can be utilized by *Ml*AT1 (an HCT), *Ml*AT2, *Ml*AT4, and *Ml*AT4 to generate their respective esters with *p*-coumaroyl-CoA or caffeoyl-CoA at varying degrees of catalytic efficiency. (**A**) The substrate pair consists of *p*-coumaroyl-CoA and shikimate, with “a”, “b”, and “c” representing three *p*-coumaroyl shikimate isomers. (**B**) The substrate pair consists of *p*-coumaroyl-CoA and 4-hydroxyphenyllactate, where “e” represents *p*-coumaroyl-4’-hydroxyphenyllactate molecule, while “d” may be an isomer with an unknown structure. (**C**) The substrate pair consists of caffeoyl-CoA and shikimate, with “f”, “g”, and “h” representing three caffeoyl shikimate isomers. (**D**) The substrate pair consists of *p*-coumaroyl-CoA and quinate, with “i”, “j”, and “k” representing three *p*-coumaroyl quinate isomers. Control groups: substrate pairs and buffer; enzyme groups: substrate pairs, enzymes, and buffer. Structural formulas in black represent acyl donors, whereas red formulas represent acyl acceptors. LC-MS was used to detect products, molecular ion peaks were extracted in ESI-mode, and fragmentation of the ions assisted in the qualitative analysis.

The results revealed that different activities were exhibited by *Ml*AT1, *Ml*AT2, *Ml*AT4, and *Ml*AT6 when incubated with different substrate pairs (Figure 2). An intriguing observation is that *Ml*AT1 and *Ml*AT6 exhibit nearly indistinguishable activity, despite belonging to distinct clades. They efficiently facilitated the transfer of acyl groups from acyl donors to shikimate or quinate, resulting in three *p*-coumaroyl shikimate isomers, three caffeoyl shikimate isomers, and three *p*-coumaroyl quinate isomers in our tests (Figure 2A,C,D). Moreover, they also catalysed the formation of *p*-coumaroyl-4’-hydroxyphenyllactate (a precursor of RA) by accepting the substrate pair consisting of *p*-coumaroyl-CoA and 4-hydroxyphenyllactate, as confirmed through precise molecular ion mass and tandem mass spectrometry (MS^2^) analysis (Figure 2B), in accordance with previous studies [31,32,33]. In the case of *Ml*AT2, only trace products were detected via mass spectrometry spectrum when using *p*-coumaroyl-CoA as the acyl donor. However, it readily accepted caffeoyl-CoA and shikimate to generate three isomeric forms of caffeoyl shikimate (Figure 2C). *Ml*AT4 exhibited a preference for transferring the acyl group from *p*-coumaroyl-CoA to shikimate to form an isomer type of *p*-coumaroyl shikimate (Figure 2A). The regional selectivity of this phenomenon appears to be more pronounced compared with its counterparts. It also displayed some ability in accepting other substrate pairs, such as promoting the conversion of *p*-coumaroyl-CoA and 4-hydroxyphenyllactate into an unknown isomer of *p*-coumaroyl-4’-hydroxyphenyllactate (Figure 2B), catalysing the generation of either *p*-coumaroyl-3’,4’-dihydroxyphenyllactate or its isomer when reacting with *p*-coumaroyl-CoA and salvianolic acid A (Figure S1), and facilitating the production of various forms of caffeoyl-shikimates by utilizing caffeoyl-CoA and shikimate. Regarding *Ml*AT6, it exhibited comparable activities to *Ml*AT1 (Figure 2) by accommodating various substrate pairs resulting in diverse enzyme-catalyzed products.

Overall, in the aforementioned activity tests, *Ml*AT1 was confirmed to be an HCT enzyme, while phylogenetically closer homologous proteins to RAS, such as *Ml*AT2, *Ml*AT4, and *Ml*AT6, also exhibited significant HCT activity. Additionally, all enzymes demonstrated detectable RAS activity, suggesting potential promiscuous activities.

### 2.3. Homology Modelling Revealed That MlAT6 Shares Multiple Active Residues Both with HCT and RAS in the Catalytic Cavity

In the aforementioned experimental tests, we observed a fascinating phenomenon in which *Ml*AT1 and *Ml*AT6, despite sharing only 51% amino acid sequence identity, exhibited identical catalytic functions. They not only demonstrate superior HCT activity, but also possess remarkable RAS catalytic capabilities. As a typical HCT enzyme with clear evolutionary status and function, the active residues in *Ml*AT1 catalytic pockets are basically consistent with other identified HCTs (Figure S2) [28]. However, the underlying catalytic principle of *Ml*AT6 is still unknown. To gain deeper insights into the binding and catalysis mechanisms of *Ml*AT6, we further investigated these processes at the molecular level.

**Figure 3:**
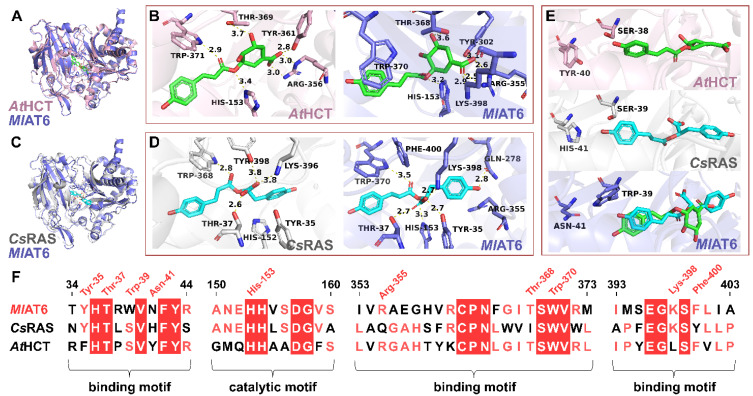
Structural comparison and sequence alignment of *Ml*AT6 with *At*HCT and *Cs*RAS. (**A**) Structure comparison of *Ml*AT6 (the docking model, purple) and *At*HCT (the crystal structure, pink). (**B**) The interaction of *p*-coumaroyl-5-O-shikimate with *At*HCT and *Ml*AT6. In *At*HCT, Arg-356 forms double salt bridges with the carboxyl group of shikimate moiety, while Tyr-361 and Thr-369 form hydrogen bonds with the ligand. In the case of *Ml*AT6, Tyr-302, Arg-355, and Lys-398 interact with the carboxyl moiety of *p*-coumaroyl-5-O-shikimate, while Thr-368 forms a hydrogen bond with the hydroxyl group. (**C**) Structural comparison of *Ml*AT6 (the docking model, purple) and *Cs*RAS (the crystal structure, grey). (**D**) The interaction of *p*-coumaroyl-4′-hydroxyphenyllactate with *Cs*RAS and *Ml*AT6. Tyr-35, Thr-37, Lys-396, and Tyr-398 in *Cs*RAS serve as potential interaction sites surrounding the carboxyl group of *p*-coumaroyl-4′-hydroxyphenyllactate, while in *Ml*AT6, Tyr-35, Thr-37, and Lys-398 act together on the ligand. In addition, Gln-278 in *Ml*AT6 is prone to forming a hydrogen bond with phenolic hydroxyl groups. (**E**) Potential residues in *Ml*AT6, *Cs*RAS, and *At*HCT that may be implicated in indirect interactions with acyl donors. (**F**) Three primary binding motifs and a catalytic motif in *Ml*AT6, *Cs*RAS, and *At*HCT. Regions highlighted in red with white text represent sequence identity among the three enzymes, while regions with light red text represent sequence identity between each two of three. Additionally, in the above catalytic pockets, Trp and His are active residues associated with the catalytic cycle, conservatively present in all plant BAHD acyltransferases. The *p*-coumaroyl-5-O-shikimate is marked in green, and the *p*-coumaroyl-4′-hydroxyphenyllactate is in cyan.

The protein structure models of *Ml*AT6 were generated using the AlphaFold2 online server [34]. The molecular model revealed that *Ml*AT6 possessed an active pocket located at the intersection of two pseudosymmetrical domains, consistent with the characteristic features of BAHD acyltransferases [35,36,37]. Subsequently, virtual docking experiments were performed to dock a *p*-coumaroyl-4’-hydroxyphenyllactate molecule and a *p*-coumaroyl shikimate molecule into the active cavity of *Ml*AT6. The resulting models from molecular docking were compared with crystal structures of *Cs*RAS (PDB code: 6MK2) [15] and *At*HCT (PDB code: 5KJU) [24] for validation purposes (Figure 3A,C). Our findings demonstrated that, in addition to the conserved active residues involved in the catalytic cycle shared by all BAHD acyltransferases, such as catalytic histidine (His-152 in *Cs*RAS, His-153 in *At*HCT, and His-153 in *Ml*AT6), as well as the tryptophan residue responsible for stabilizing negatively charged tetrahedral transition-state intermediates (Trp-368 in *Cs*RAS, Trp-371 in *At*HCT, and Trp-370 in *Ml*AT6), according to previous studies [38], multiple active sites within acyl receptor binding domain have been identified between *Ml*AT6, *Cs*RAS, and *At*HCT.

For example, in *Cs*RAS, there are four polar residues (Tyr-35, Thr-37, Lys-396, and Tyr-398) that potentially serve as binding sites for the carboxyl group of 4-hydroxyphenyllactate (Figure 3D) and are conserved in Lamiaceae RASs. Molecular docking analysis suggested that *Ml*AT6 also has a similar acyl acceptor recognition mechanism. Tyr-35 and Thr-37 in *Ml*AT6 may interact with the 4-hydroxyphenyllactate unit through hydrogen bonds, while Lys-398 may form a salt bridge with the carboxylate group of *p*-coumaroyl-4’-hydroxyphenyllactate (Figure 3D). There was only one non-polar substitution at those four positions (Phe-400 in *Ml*AT6).

Similarly, *Ml*AT6 also possesses multiple active residues similar to *At*HCT, such as Arg-355 and Thr-368 in *Ml*AT6 and Arg-356 and Thr-369 in *At*HCT (Figure 3B), which are favourable for binding shikimate molecules [28]. In the docking model of *Ml*AT6, Arg-355 appeared to form multiple salt bridges with the carboxyl group of the shikimate unit (Figure 3B). It has been confirmed that arginine at the same position as HCT is a prominent site for recognizing specific acyl acceptors because it can build stable double salt bridges with the carboxyl group of a shikimate or its analogues [28,38]. Additionally, compared with *At*HCT, *Ml*AT6 contains certain extra residues likely involved in contact with the shikimate unit, including Tyr-302 and Lys-398.

As previously mentioned, *Ml*AT6 shares multiple active residues both in the acyl receptor binding region of the active pocket with *Cs*RAS and *At*HCT. In contrast, the binding domains of acyl donors exhibit several substitutions of active residues that may have indirect contact with acyl-CoA thioesters [28,38] (Figure 3E). Specifically, two acidic residues, Ser-38 and Tyr-40 in *At*HCT, are substituted by two basic residues, Trp-39 and Asn-41 in *Ml*AT6, while at the same place as *Cs*RAS, there is the acidic residue Ser-39 and the basic residue His-41.

Moreover, by identifying these key residues in the active pockets of *Ml*AT6, *Cs*RAS, and *At*HCT, we obtained three binding motifs along with one catalytic motif (Figure 3F). We observed that a majority of the members of *Ml*ATs universally exhibited significant characteristics of both HCT and RAS at these specific motifs, especially in the acyl-acceptor-binding region (Figure S3), which indicates that *Ml*AT6 probably has a specific acyl acceptor recognition mechanism similar to both RAS and HCT mechanisms, providing an explanation for its concurrent RAS and HCT activity. In subsequent experiments, we introduced mutations to two key active residues (Arg-355 and Lys-398) in the *Ml*AT6 active pocket. In vitro activity assays demonstrated their indispensability for protein function, as mutation of either R355A or K398L resulted in a complete loss of catalytic activity (Figure S4).

### 2.4. The Narrow Catalytic Cavities of MlATs May Impose Constraints on Substrate Binding

**Figure 4:**
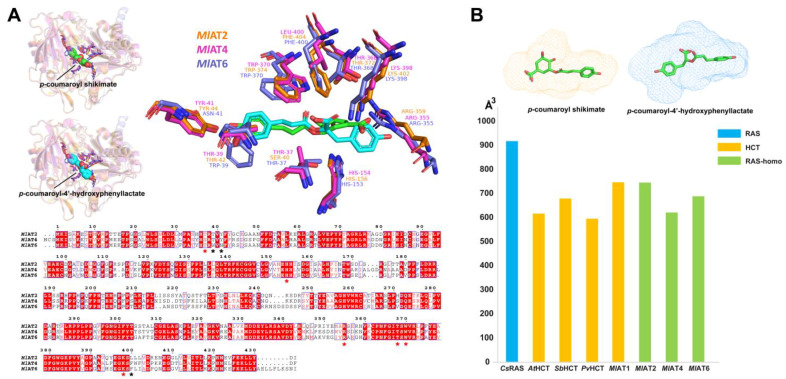
(**A**) Structural comparison and multiple sequence alignment of *Ml*AT2, *Ml*AT4, and *Ml*AT6. They are conserved in the acyl receptor binding domain and exhibit similar molecular characteristics overall. Regions highlighted in red with white text represent sequence identity among the three enzymes. The active residues in the catalytic centre are indicated with an asterisk; red indicates good sequence identity; and black indicates that they are not conservative. (**B**) Calculated volumes of proteins’ active cavities. The volume of the active pocket of *Cs*RAS was significantly larger than that of HCTs and *Ml*ATs. It should be pointed out that the *Cs*RAS and HCTs used in the calculation are crystal structures, whereas *Ml*ATs correspond to structural models.

The structural models of *Ml*AT2 and *Ml*AT4 were obtained using the Alphafold2 program, and their comparison with *Ml*AT6 was performed (Figure 4A). In terms of the distribution of active residues in catalytic pockets, *Ml*AT2 and *Ml*AT4 are similar to *Ml*AT6, especially in acyl receptor binding regions. However, in the acyl donor binding sites, two basic residues (Trp-39 and Asn-41 in *Ml*AT6) were substituted by two acidic residues (Thr-39 and Tyr-41 in *Ml*AT2, Thr-42 and Tyr-44 in *Ml*AT4).

Although phylogenetically and molecularly close to RAS, the ability of these *Ml*ATs to accept 4-hydroxyphenyllactate or 3,4-dihydroxyphenyllactate was found to be insignificant based on the available data. To investigate the potential constraints on their catalytic activity, we calculated the active pocket volumes for several protein crystals and models. The results revealed an expanded *Cs*RAS active pocket with a cavity volume exceeding 900 Å^3^ compared with an average active pocket volume of less than 700 Å^3^ for HCTs or *Ml*ATs (Figure 4B).

### 2.5. Tandem Duplication of the HCT Gene Serves a Prerequisite for the Emergence of RAS

**Figure 5:**
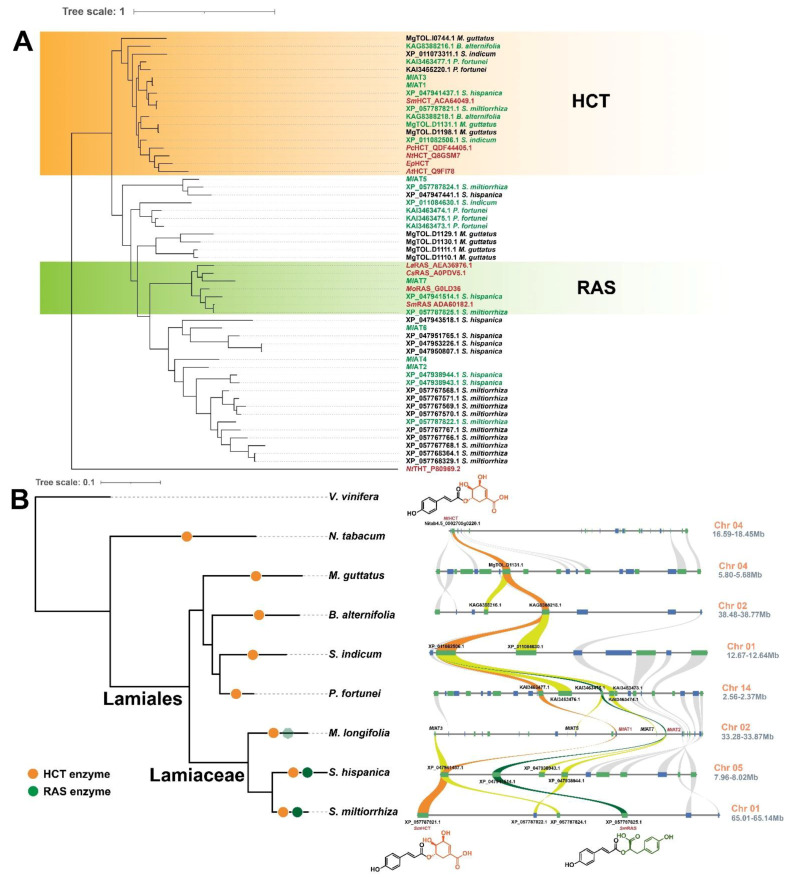
Expansion and diversification of the HCT enzyme family in plants. (**A**) Phylogenetic analysis of HCT and its homologous enzymes in Lamiaceae and its relatives. *Nt*THT (Hydroxycinnamoyl-CoA: tyramine N-hydroxycinnamoyltransferase) as the outgroup. Enzymes highlighted in red indicate verified enzymes that have undergone functional validation and serve as a point of reference. Enzymes highlighted in green indicate those that are located within the collinear block of chromosomes. (**B**) Microsyntenty analysis of HCT regions and RAS regions. Red gene names signify that the gene has undergone functional validation, while black denotes the annotation name. The interconnections among all HCT genes on the local chromosomal region are visually emphasized in orange, while the green lines depict the genetic correlation of the *Sm*RAS gene. The links to other homologous genes of HCT are highlighted in a yellow-green colour. Enzymes in the species tree are depicted with solid circles of distinct colours, where orange represents HCT enzymes and green represents RAS enzymes. “Chr” is an abbreviation for chromosome.

The available sequence and functional data supported a potentially close evolutionary relationship between *Ml*ATs and the existing Lamiaceae RASs and HCTs. To gain further insights into these aspects, we conducted an extensive search for additional evidence from plant genomes. Previous studies have demonstrated the conservation of catalytic properties and physiological function of HCT throughout the 500 million years of embryophyte evolution [18]. This suggests that many existing HCT homologous proteins, including RAS and *Ml*ATs, may have originated from ancestral HCT duplications. To validate this hypothesis, we investigated plant genomes of Lamiaceae and its relatives to identify all genes homologous to HCT. The resulting encoding proteins were aligned with several well-characterized hydroxycinnamoyl CoA transferases for phylogeny reconstruction (Figure 5A). The phylogenetic analysis revealed a strongly supported clade consisting of one or more members from each species at the root, representing the HCT clade. Other proteins formed distinct clades as sister groups to HCT, with the RAS family being one of them.

Subsequently, a species tree of Lamiaceae and its sister families was constructed, and the syntenic segments harbouring HCT genes on the chromosomes of these species were identified (Figure 5B). The findings demonstrated that the tandem duplication of HCT synthesis genes in certain species led to the presence of multiple homologous genes. The *Ml*AT synthesis genes on chromosome 2 of the *Mentha longifolia* genome are arranged in collinear regions, where *Ml*AT1 and *Ml*AT2 exhibit close evolutionary relationships to HCT and RAS, respectively. Consistent with previous hypotheses, RAS synthesis genes in Lamiaceae were derived from the duplication and differentiation of the ancestral HCT gene, and the genetic correlation of *Sm*RAS (Accession: ADA60182.1) can be traced back as distant as *Nt*HCT (Accession: Q8GSM7.1) in tobacco.

To be clear, we did not find intraspecific and interspecific collinear associations of RAS homologous proteins on chromosomes 7 and 9 of *Menta longifolia*. These homologous proteins at other chromosomal locations may have been acquired through dispersive duplication or other mechanisms, but we currently lack sufficient insights into these processes. Moreover, our study did not identify a functionally and evolutionarily intact *Ml*RAS synthesis gene in *Menta longifolia*. The closest evolutionary relative to known RAS genes is *Ml*AT7; however, it displays an incomplete genic structure, resembling pseudogenes.

## 3. Discussion

Plants employ various specialized enzymes to synthesize a wide range of structurally and functionally diverse metabolites, enabling them to adapt to changing environmental stresses. Investigating the origin and evolution of these specialized metabolic enzymes has long been a crucial aspect in plant evolutionary research. Up to now, extensive efforts have been dedicated to comprehend the generation and development of plant-specialized metabolism [39,40,41], which not only contributes to enhancing our understanding of plant evolution, but also aids in elucidating the existing constraints on metabolic function and devising improvement strategies [42,43]. In recent years, there has been increased focus among biologists on the evolution of functional diversity in plant-specialized enzyme families that contribute to metabolic diversity [44,45,46]. However, understanding these processes remains challenging due to the parallel elimination of most evolutionary intermediates by distinct lineages during extensive historical development [47]. The reality is that our exploration of evolutionary footprints is limited to only a small fraction of the available historical phylogenetic samples. Fortunately, significant advancements in plant genome sequencing over the past decade have provided a substantial volume of sequence data, enabling a comprehensive understanding of enzyme evolutionary pathways by connecting related yet discrete biological features. Herein, we employed an interdisciplinary approach integrating chemical, structural, functional, and sequence data within a phylogenetic framework to gain some insights into the evolutionary patterns exhibited by plant-specialized metabolic enzymes.

In general, homologous proteins from closely related species serve as important reference materials for studying the evolutionary patterns of metabolic enzymes [48]. By analysing the genome sequences of *M. longifolia*, we successfully identified one HCT (*Ml*AT1) and several RAS homologous enzymes. In vitro functional verification and molecular modelling suggest that they possess catalytic activities and molecular features similar to both HCT and RAS (Figure 2, Figure 3 and Figure 4). Interestingly, despite their closer phylogenetic affinity to Lamiaceae RASs (Figure 1B), they appear to demonstrate a slightly stronger overall HCT function (Figure 2). Structural analysis of these enzymes’ active sites further revealed an acyl-acceptor-binding region resembling both HCT and RAS (Figure 4). Overall, this group of enzymes exhibits a convergence of molecular mechanisms between HCT and RAS, suggesting their unique evolutionary position. Investigation into the genomes of Lamiaceae and closely related species has significantly expanded the populations of homologous enzymes for both RAS and HCT (Figure 5A). Comparative genomic analysis revealed that RAS homologous genes on chromosome 2 of *Mentha longifolia* emerged through tandem duplication events of HCT genes, with *Ml*AT2 exhibiting a close genetic relationship with the *Sm*RAS (Figure 5B). Despite the convergence in sequence and structure, *Ml*AT2 exhibits limited RAS activity (Figure 2). The genuine RAS synthesis gene was absent in *Mentha longifolia*.

The production and accumulation of some beneficial compounds can confer a competitive advantage in specific ecological niches [49]. Thus, a universally observed phenomenon is that evolution readily selects those promiscuous activities with biological adaptive advantages from existing proteins as the starting point for new functions [50,51,52,53]. In previous reports, RA has been confirmed to play an important role in plant defence, including photoprotection and insect resistance [54,55]. We inferred that the emergence of RAS and its homologous enzymes in Lamiaceae can likely be attributed to the substrate permissiveness of progenitor HCTs towards 4-hydroxyphenyllactate and its analogues. The resulting RA or its analogues might have been favoured by plants for ecological benefits, leading to the subsequent evolution of favourable enzyme activities. It should be emphasized that a trade-off often exists between the enhancement of novel functions and the retention of original functionalities, as these processes typically involve distinct molecular mechanisms. In this case, gene duplication is considered an indispensable prerequisite that creates physical conditions for adaptive protein evolution [56]. Gene tandem duplications of HCT in Lamiaceae have not only expanded this enzyme group, but also relieved restrictive pressure on nucleotide sequences, allowing the accumulation of mutations. In general, the forces of natural selection tend to maintain mutations that confer substantial adaptation advantages, resulting in a collective change in a specific direction at the sequence level. As a result, it is not surprising that these duplicated HCT genes exhibit sequence characteristics that progressively approach the RAS function throughout evolution. Previous studies have shown that the functional specialization of members of the BAHD acyltransferase family may result in sequence variants that are concentrated in specific motifs, rather than globally distributed variants throughout the enzyme structure [57]. Our study also observed this phenomenon and emphasized the connections and distinctions between these homologous enzymes with HCT and RAS in binding and catalytic motifs. As a clade that preserves evolutionary traits, they provide a link for understanding functional differentiation between HCT and RAS.

However, it is worth noting that the majority of these ultimately undergo loss rather than adaption, since evolutionary outcomes are often accidental [58,59]; this aligns with the weaker function observed in some enzymes. Despite not being in an optimal evolutionary form, enzyme genes can persist within a population if they are capable of producing specific compounds that offer adaptive advantages to plants [60]. An intriguing concept is that these non-specific homologous enzymes may possess enhanced evolutionary plasticity as a result of lacking strict functional constraints, particularly regarding functional promiscuity and its potential for enhancing adaptive evolution [50,61,62]. Notably, our study was unable to ascertain the ancestral origin of all RAS homologous proteins discovered in peppermint. There are various types of gene duplication events caused by genetic aberrations, including replication slippage, retrotransposition, ectopic recombination, aneuploidy, and polyploidy [56]. Despite their associations with HCT and RAS based on sequence, structure, and function, we lack qualitative evidence regarding the source of other proteins scattered throughout the genome, including *Ml*AT4 and *Ml*AT6. They could have been acquired through decentralized repetition mechanisms, or might have functioned as enzymes assuming alternative roles.

Furthermore, in addition to plants belonging to the Lamiaceae and Boraginaceae families, sporadic traces of the RA chemotype have also been found in various taxa of flowering plants, as well as some early plant groups such as hornworts and ferns [13,14,63]. While existing evidence supports the independent evolution of RA biosynthesis in different plant lineages, our study suggests that enzyme promiscuity may also contribute to the accumulation of RA, which is consistent with the trace amounts observed in certain plant taxa. Overall, the findings of our study offer novel evidence regarding the origin and evolutionary trajectory of RAS in Lamiaceae, achieved through a comprehensive identification process of RAS homologous enzymes. Additionally, our research provides some insights into the intricate mechanisms underlying the metabolism and accumulation of RA in *Mentha longifolia*.

## 4. Conclusions

In this study, we conducted a comprehensive investigation and characterization of several RAS homologues to unravel their divergence from the HCT family, leading to the emergence and enhancement of the RA metabolic pathway. Our analysis suggests that gene tandem duplications of ancestral HCTs during Lamiaceae plant evolution resulted in extensive homologous enzymes. Subsequent selection pressures likely drove them to explore new functions for enhanced biological adaptability. In this process, a small number of enzymes have completed functional divergence and acquired specific RAS functions. Most of the other enzymes eventually aggregate in the adjacent branches of the Lamiaceae RASs in phylogenetic trees, exhibiting molecular characteristics similar to RAS but with less activity. As an offshoot to the RAS developmental trajectory, the analysis of these enzymes has provided a glimpse into their evolution.

Our work enhances understanding of the developmental trajectory from HCT to RAS in Lamiaceae by elucidating functional and structural features shared among different homologous enzymes. This provides some insights into the evolution of specialized metabolic enzymes in plants. The observed evolutionary pattern reflects a natural order resulting from random mutations guided by function to meet adaptive needs. The study also provides a novel case in favour of the fundamental theory of enzymatic evolution. The expansion of enzyme families weakens functional constraints and creates favourable conditions for new enzyme emergence, shaping a diverse and adaptable evolutionary landscape. The acquisition of new functions does not necessarily disrupt old mechanisms completely; there may be an adaptive transitional stage before switching occurs. In future research, increasingly sophisticated experimental techniques and enriched omics data will provide opportunities for the better understanding, more efficient utilization, and more precise development of these enzymes.

## 5. Materials and Methods

### 5.1. Genome Mining

Genome data of *Mentha longifolia*. (Horse mint) are stored in the Mint Genomics Resource (http://langelabtools.wsu.edu/mgr/ (accessed on 7 October 2022)). The database provides an online BLAST [64] server. The TBLASTN search was used to perform a homologous search for RAS, identifying database sequences encoding proteins similar to the query. Proteins encoded by these candidate genes were preliminarily annotated using the NCBI database (https://www.ncbi.nlm.nih.gov/ (accessed on 8 October 2022)) and the UniProtKB/Swiss-Prot database (https://www.uniprot.org (accessed on 8 October 2022)).

The BLAST search was used to investigate orthologous genes of HCT in the genomes of Lamiaceae and related species, including *Buddleja alternifolia* (GCA_019426215.1), *Mimulus guttatus* (MguttatusTOL_551_v5.0), *Paulownia fortune* (GCA_019321725.1), *Salvia hispanica* (GCF_023119035.1), *Sesamum indicum* (GCF_000512975.1), and *Salvia miltiorrhiza* (GCF_028751815.1). These plant genomes were downloaded from Phytozome (https://phytozome-next.jgi.doe.gov/ (accessed on 12 January 2024)) and NCBI. In addition, the HCT gene (*Nt*HCT) in the genome of *Nicotiana tabacum* was searched and located, aiming to provide a reference for the evolutionary origins of HCT homologous genes in other species. The tobacco genome (Nitab-v4.5) was downloaded from Sol Genomics Network, https://solgenomics.net/ (accessed on 13 January 2024).

### 5.2. Phylogenetic Analysis of Candidate Homologous Genes of RAS

The amino acid sequences were aligned using the MUSCLE algorithm [65]. Subsequently, phylogenetic analyses were conducted in Mrbayes (v3.2.6) [66] with the following parameters: average standard deviation = 0.1; minimum generation = 500,000; maximum generation = 0 (∞); and detection every 5000 generations. Other BAHD-AT sequences for analysis can be downloaded from the NCBI database. Enzymes used to build the phylogenetic trees can be queried in Table S1.

### 5.3. Molecular Biology

The CDSs of *Ml*AT1, *Ml*AT2, *Ml*AT4, and *Ml*AT6 were synthesized through the gene service company (Tsingke, Nanjing, China) and inserted into the pET-28a(+) plasmid with a 6×His tag at the N-terminus (completed by the company). All sequences were optimized to make them more suitable for expression in *E. coli* (the process of codon optimization does not alter the amino acid sequence). Additionally, *Ml*AT1 and *Ml*AT4 were further inserted into the pET-28a(+)-SUMO plasmid with a 6×His tag and a SUMO tag at the N-terminus to promote protein solubility. The CDSs were prepared through PCR amplification from the pET-28a(+)_*Ml*AT1 and pET-28a(+)_*Ml*AT4 plasmids using I-5™ 2X High-Fidelity Master Mix (MCLAB, Beijing, China) and then cloned into the target vector using the Uniclone One-Step Seamless Cloning Kit (Genesand, Beijing, China). Mutant plasmids of R355A, K398L, and R355A+K398L were prepared from the pET-28a(+)_*Ml*AT6 plasmid by designing primer pairs at specific mutant sites and using the multi-gene fragment recombination strategy.

Plasmids were linearized using FastDigest restriction enzymes purchased from Thermo Scientific as follows: pET-28a(+) was linearized with Nhe Ⅰ and BamH Ⅰ; pET-28a(+)-SUMO was linearized with Nhe Ⅰ and BamH Ⅰ.

The primers utilized in the experiments are listed in Table S2. The coding sequences (CDSs) of *Ml*ATs are presented in Information S1, while the optimized sequences are available in Information S2. Additionally, the mutation sequences of MlAT6 can be found in Information S3.

### 5.4. Recombinant Protein Expression and Purification

The plasmids of pET-28a(+)-SUMO_*Ml*AT1, pET-28a(+)_*Ml*AT2, and pET-28a(+)-SUMO_*Ml*AT4 were transformed into pGro7/BL21(DE3) chaperone competent cells; pET-28a(+)_*Ml*AT6 were transformed into Rosetta (DE3) competent cells. The cells were grown in terrific broth medium at 37 °C for 2–3 h, followed by the induction of expression with 0.1 mM IPTG at 18 °C for 16–18 h. For the strains of pGro7/BL21(DE3), an additional 1 mg/mL *L*-arabinose was added to the medium to induce the expression of chaperone proteins. After centrifugation at 9000× *g* for 30 min, the cells were resuspended in lysis buffer (50 mM Tris-HCl, 300 mM NaCl, 25 mM imidazole, pH 8.0) and lysed using an Ultrasonic Homogenizer (Scientz, Ningbo, China) at 4 °C. The lysate was clarified by centrifugation at 15,000× *g* for 1 h at 4 °C and filtered to remove insoluble cell fragments. The clarified lysate was immediately affinity-purified using a HisTrap FF crude 5 mL column (Amersham Biosciences, Slough, UK) prepacked with precharged Ni Sepharose™ 6 Fast Flow for the preparative purification of histidine-tagged recombinant proteins through immobilized metal affinity chromatography. A large amount of binding buffer (50 mM Tris-HCl, 1 M NaCl, 25 mM imidazole, PH 8.0) was used to wash off the heteroproteins. Elution buffer (50 mM Tris-HCl, 200 mM NaCl, and 300 mM imidazole, PH 8.0) was used to wash off the histidine-tagged recombinant proteins. Recombinant proteins containing a SUMO tag needed to have the label removed by using SUMO protease digestion. The processed proteins did not have an affinity for nickel ions; they could flow out with the binding buffer. The collected solution was dialyzed overnight at 4 °C in dialysis buffer (50 mM Tris-HCl, 50 mM NaCl, and 10% glycerol) to remove imidazole. The dialyzed protein was concentrated to 5–20 mg/mL in an Amicon Ultra-15 mL 10 kDa Centrifugal Filter Unit (Millipore, Burlington, MA, USA), flash-frozen in liquid nitrogen, and stored at −80 °C until use. The preparation of *Ml*AT6 mutant proteins followed the same protocol as that for the *Ml*AT6 proteins.

### 5.5. Enzyme Assays

Recombinant proteins were taken out from −80 °C and thawed in the ice. The thawed solution needed to have glycerol removed in order to reduce side reactions. This was performed by using an Amicon Ultra-0.5 mL 10 kDa Centrifugal Filter Unit (Millipore) to replace the storage buffer with the reaction buffer, which consisted of 50 mM Tris-HCl, pH 8.0, 1 mM DTT, and 0.5 mM ascorbic acid. Each protein was diluted to 5 mg/mL.

The enzymatic activity of recombinant proteins (*Ml*AT1, *Ml*AT2, *Ml*AT4, and *Ml*AT6) was determined by incubating protein fractions in a total volume of 125 μL, consisting of 110 μL reaction buffer, 2.5 μL 100 mM acyl donor (*p*-coumaroyl-CoA or caffeoyl-CoA), 2.5 μL 10 mM acyl acceptor (4-hydroxyphenyllactate, 3,4-dihydroxyphenyllactate, shikimate, or quinate), along with 10 μL recombinant proteins. All reactions were carried out at 30 °C for 3 h, and then 20 μL 6 N HCl was added to terminate them. The reaction products were extracted three times with 500 μL ethyl acetate each time, and then evaporated to remove the solvent. The extracts were redissolved in 150 μL 50% methanol and filtered for LC-MS analysis. We detected significant hydrolysis of hydroxycinnamoyl-CoA under the catalysis of enzymes in tests; therefore, we did not explore the enzyme kinetics of the acyl transfer reaction.

The enzymatic activity of *Ml*AT6 and its mutant proteins were determined in total volumes of 50 μL, consisting of 40 μL reaction buffer, 1 μL 100 mM *p*-coumaroyl-CoA, 1 μL 10 mM shikimate, and 8 μL recombinant proteins. The reaction was carried out at 28 °C for 2 h, and then 50 μL MeOH was added to terminate. The solution was centrifuged at 17,000× *g* for 15 min, and then the supernatant was used for LC-MS analysis. Each reaction was repeated three times.

(Supplementary instruction: These reaction conditions, including the reaction time, temperature, and termination conditions, were all established based on the hydroxycinnamoyl transferase reactions reported in the literature. Additionally, we conducted a time gradient analysis of the reaction and observed consistent production of multiple isomers, regardless of whether the reaction duration was 30 min or 3 h.)

### 5.6. UHPLC-Q-Exactive Focus Mass Spectrometry

LC was performed on a Dionex UltiMate 3000 UHPLC system (Thermo Fisher Scientific, Waltham, MA, USA) using Milli-Q water (solvent A) and methanol (solvent B). Samples, including the groups of *p*-coumaroyl-CoA and shikimate, *p*-coumaroyl-CoA and 4-hydroxyphenyllactate, and caffeoyl-CoA and shikimate, were analysed using an Eclipse XDB C18 80 Å column (length 250 mm, diameter 3 mm, particle size 5 μm, Agilent, Santa Clara, CA, USA). The elution gradient was as follows: 10–30%B for 5 min, 30–70%B for 5 min, 70–90%B for 6 min, 90%B for 2 min, 90–10%B for 2 min, and 10%B for 5 min at a flow rate of 1 mL/min. For samples of the group of *p*-coumaroyl-CoA and quinate, the elution gradient was modified as follows: 10–50%B for 5 min, 50–70%B for 7 min, 70–90%B for 2 min, 90–10%B for 1 min, and 10%B for 5 min at a flow rate of 1 mL/min. The same conditions were applied to the samples of hydrolysis reactions.

Additionally, samples of *Ml*AT6 and its mutant proteins were analysed using a ZORBAX 300SB-C8 column (length 150 mm, diameter 4.6 mm, particle size 5 μm, Agilent), equipped with a protective column (Phenomenex SecurityGuard KJ0-4282, Guangzhou, China). The elution gradient was set as follows: 10%–30%B for 3 min, 30%–90%B for 12 min, 90%B for 5 min, 90%–10%B for 1 min, and 10%B for 4 min at a flow rate of 0.3 mL/min.

All compounds were detected using a high-resolution Q-Executive Focus Orbitrap Mass Spectrometer (Thermo Fisher Scientific), operating in negative ionization mode (ESI-) with a full scan range of 120–1200 *m/z*. All data were analysed using the Xcalibur 4.0 Qual Browser (Thermo Fisher Scientific).

### 5.7. Prediction of Protein Structural Models

The homologous models of acyltransferases were generated by submitting amino acid sequences to the AlphaFold2 Protein Structure Prediction Online Server (https://colab.research.google.com/github/sokrypton/ColabFold/blob/main/AlphaFold2.ipynb (accessed on 25 July 2022)). The process was executed using default settings, and the rank1 output was utilized for comparative structural analysis. Additionally, we compared the prediction result with those obtained from other protein structure prediction servers, namely Robetta (https://robetta.bakerlab.org/ (accessed on 6 July 2022)) and Phyre2 (http://www.sbg.bio.ic.ac.uk/~phyre2/html/page.cgi?id=index (accessed on 6 July 2022)). The root-mean-squared deviation (RMSD) values for the same protein predicted by different programs were less than 2 Å, as calculated in PyMOL (2.5.2).

### 5.8. Molecular Docking

The crystal structures of *Cs*RAS (PDB ID: 6MK2) and *At*HCT (PDB ID: 5KJU) were downloaded from Uniprot (https://www.uniprot.org/ (accessed on 12 July 2022)). All ligands were drawn using ChemBio3D Ultra 14.0 and saved in PDB format. Due to an incorrect connection between the Arg-355 and the Tyr-302 in the rank1 output of *Ml*AT6, the rank2 output was selected for docking. Docking was performed using AutoDock4, and the results were analysed using AutoDockTools (v.1.5.6). We employed a semi-flexible docking method where the protein was treated as rigid, except for Arg-355 in *Ml*AT6, which was set as a flexible amino acid residue. The grid covered the active cavity with an appropriate cube size. Docking conformations were generated using a Lamarckian genetic algorithm (LGA), and the results were ordered based on the interaction energy between the substrate and target protein, from lowest to highest. Low-energy conformations were our focus. In addition, references in the literature provided positions and conformations for some active residues of acyltransferases. All results were visualized on PyMOL, including aligned structures, measured distances, calculated RMSD, labelled residues, and figure preparation. Finally, Adobe Illustrator 2023 was used to combine and enhance these images.

### 5.9. Multiple Sequence Alignment

Multiple sequence alignments were performed and visualized in Jalview (v.2.11.2.6) using the MUSCLE algorithm with default parameters. The displayed results were enhanced using Adobe Illustrator 2023. The brighter the colour, the more conserved the site. In addition, we used ESPript 3.0 to help with visualization.

### 5.10. Calculation of Volumes of Protein Activity Pockets

The active pocket volumes of protein crystals and structural models were analysed using parKVFinder v1.0.2, with the cavity detection modes set to “ligand adjustment”, the “probe out” adjusted to 10.0, the “removal distance” set to 0.0, and all other parameters defaulted.

### 5.11. Species Tree

Orthologs were identified using OrthoFinder v2.5.4 software among nine plant species: Vitis vinifera, Nicotiana tabacum [67], Buddleja alternifolia [68], Sesamum indicum [69], Paulownia fortunei [70], Mimulus guttatus, Mentha longifolia [30], Salvia miltiorrhiza [71], and Salvia hispanica [72]. The protein sequences of single-copy orthologous genes were used to construct a phylogenetic tree.

### 5.12. Chromosomal Collinearity Analysis

The nine plant species used to construct the tree were analysed: *Buddleja alternifolia* (GCA_019426215.1), *Mimulus guttatus* (MguttatusTOL_551_v5.0), *Paulownia fortune* (GCA_019321725.1), *Salvia hispanica* (GCF_023119035.1), *Sesamum indicum* (GCF_000512975.1), *Salvia miltiorrhiza* (GCF_028751815.1), *Nicotiana tabacum* (Nitab-v4.5), and *V. vinifera* (*Vvinifera*_457_v2.1). Protein sequences and GFF/GFF3 attachments from fully sequenced genomes were downloaded from the Phytozome, NCBI, and Sol Genomics Network. Genomic collinearity was detected using MCScan (Python version) (https://github.com/tanghaibao/jcvi/wiki/MCscan-(Python-version) (accessed on 15 January 2024)) with default parameters, resulting in the construction of a library dataset that included all possible syntenic gene pairs among the nine plant genomes. Chromosomal collinearity fragments containing HCT and its homologous genes were selected for mapping and visualization.

## Data Availability

The Supplementary Materials and PDB files are available within the article. Other raw data are available on request from the corresponding author.

## Supplementary Materials

The following supporting information can be downloaded at: https://www.mdpi.com/article/10.3390/plants13040512/s1, Figure S1: *Ml*AT4 has RAS catalytic potential; Figure S2: The homology model of *Ml*AT1; Figure S3: The multiple sequence alignment of HCTs, RAS-homo proteins in *Menta longifolia*, and Lamiaceae RASs; Figure S4: Validation of two key active residues; Table S1: Enzymes used for the phylogenetic tree; Table S2: PCR primers used for protein expression; Information S1: The CDS sequences of *Ml*AT1-*Ml*AT7; Information S2: The optimized sequences of *Ml*AT1, *Ml*AT2, *Ml*AT4 and *Ml*AT6; Information S3: The nucleotide sequences of *Ml*AT6 mutants.
