# Supplementary material for: Analysing a Group of Homologous BAHD Enzymes Provides Insights into the Evolutionary Transition of Rosmarinic Acid Synthases from Hydroxycinnamoyl-CoA:Shikimate/Quinate Hydroxycinnamoyl Transferases
Source: Plants (Basel). 2024 Feb 13;13(4):512. doi: 10.3390/plants13040512 (PMC10892161; doi:10.3390/plants13040512)
Supplement: Supplementary file 1 [file plants-13-00512-s001.zip › plants-2854421-supplementary.pdf]

Revealing a Cluster of Homologous Proteins Provides New Insights Into the Evolutionary Transition from hydroxycinnamoyl-CoA: shikimate/quinate hydroxycinnamoyl transferase to rosmarinic acid synthase

Jiali Zhou[1], Xiaofang Zou[1], Zixin Deng[1], Lian Duan[1*]

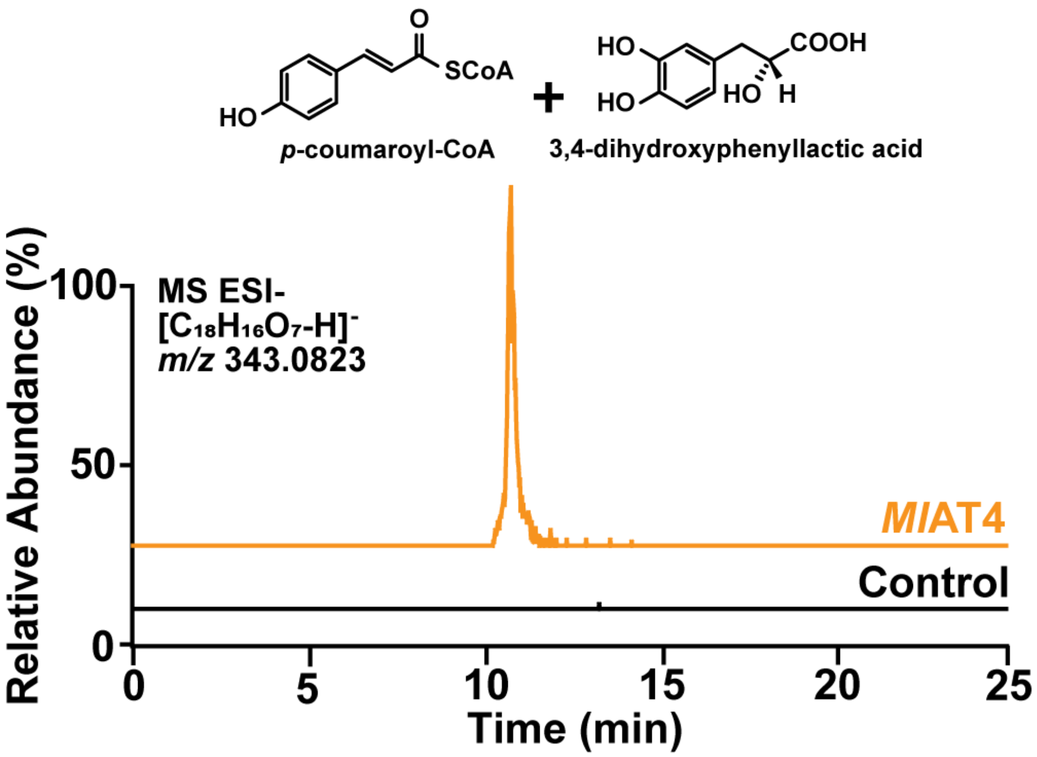

Figure S1. *Ml*AT4 has RAS catalytic potential. *Ml*AT4 has the ability to catalyze the production of a small quantity of *p*-coumaroyl-3', 4'-dihydroxyphenyllactate or its isomer by converting *p*-coumaroyl-CoA and salvianolic acid A (3,4-dihydroxyphenyllactic acid). The product was identified through mass spectrometry, with molecular ion peaks extracted while operating in negative ion mode.

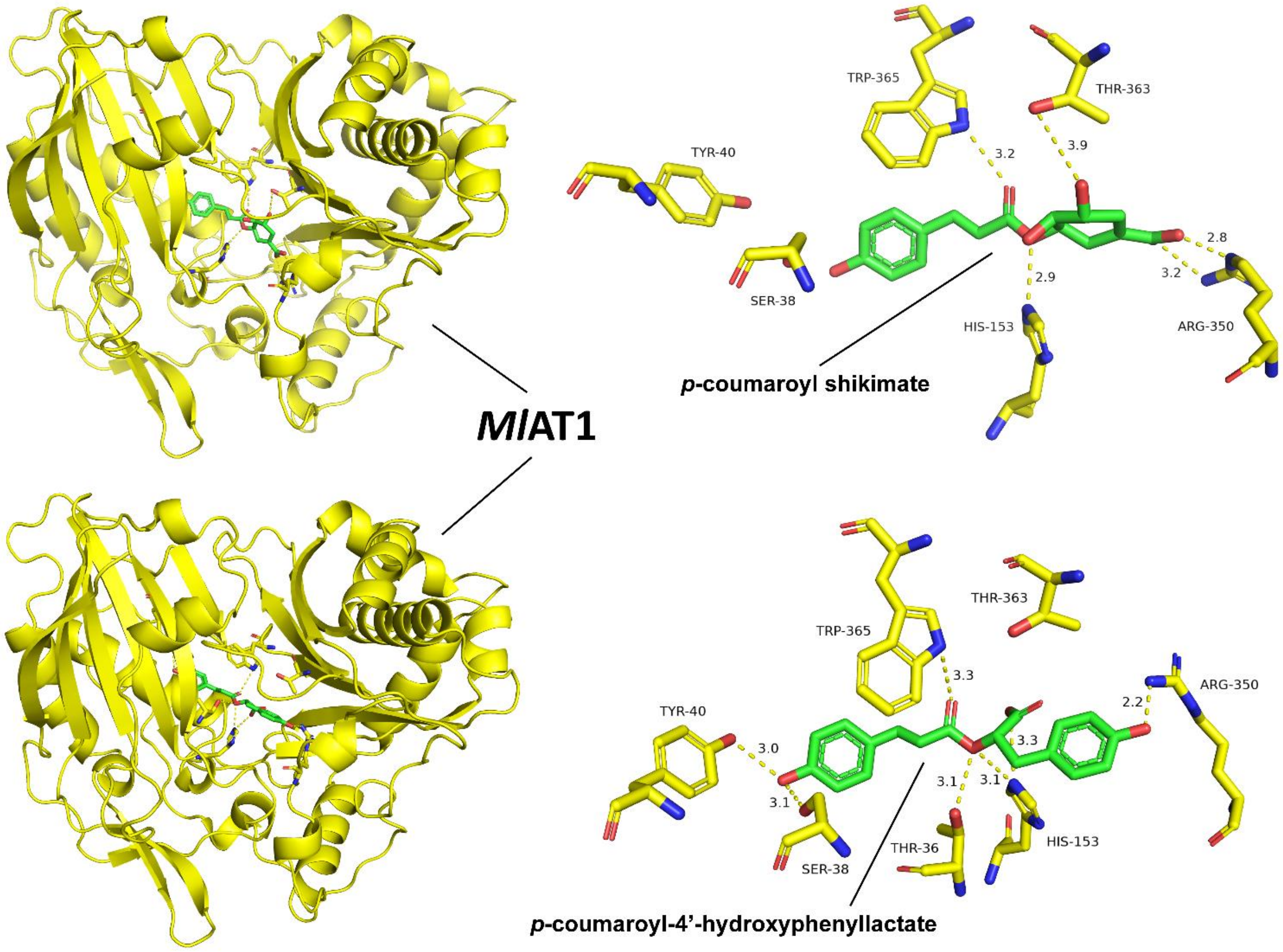

Figure S2. The homology model of *Ml*AT1. The binding mode between the enzyme and *p*-coumaroyl shikimate resembles that observed in other HCTs. Additionally, active residues in its catalytic cavity might non-specifically guide the binding of 4-hydroxyphenyllactate, resulting in promiscuous activity. Thr-36, Arg-350, and Thr-363 may all potentially provide binding sites for the 4-hydroxyphenyllactate molecule. Ligand molecules are labeled in green, and residues are displayed in yellow.

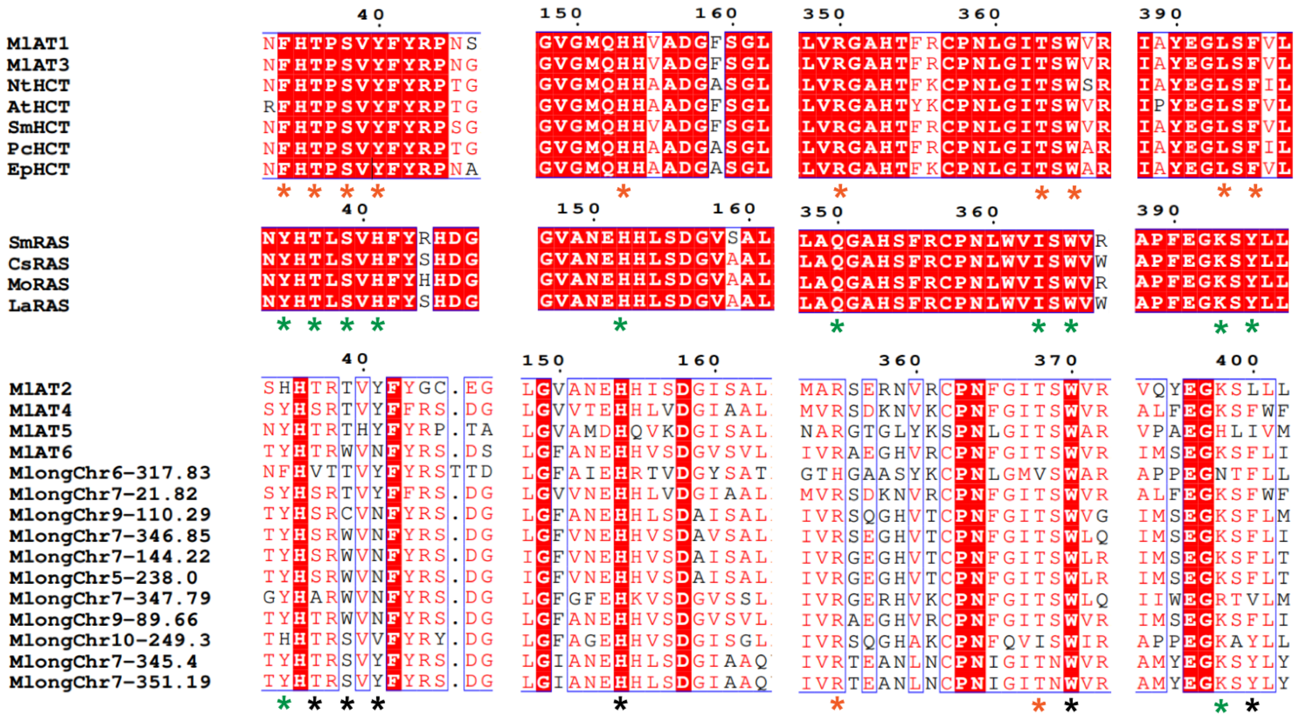

Figure S3. The multiple sequence alignment of HCTs, RAS-homo proteins in *Menta longifolia*, and Lamiaceae RASs. Active residues in the catalytic pocket were indicated by asterisks. Orange asterisks denote active residues conserved in HCT, while green asterisks represent active residues conserved in RAS. The residues associated with acyl acceptor binding in RAS-homo exhibit significant dual characteristics of both HCT and RAS.

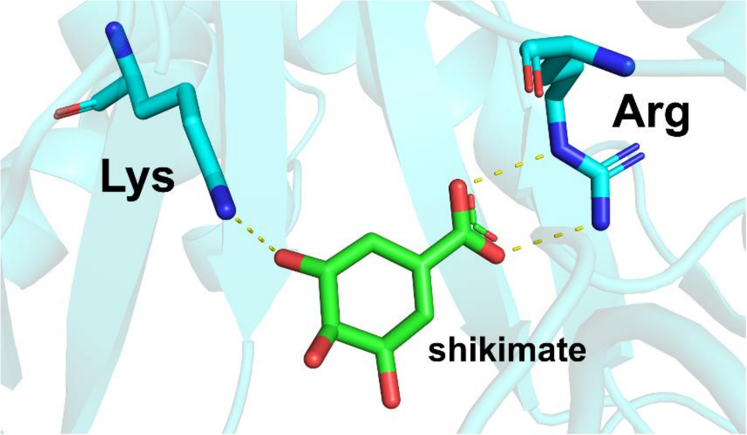

	p-coumaroyl shikimates
*Ml*AT6	100%
R355A	0%
K398L	0%
R355A+K398L	0%

Figure S4. Validation of two key active residues. Mutation of either of two active residues (the arginine residue Arg-355 and the lysine residue Lys-398) in *Ml*AT6 results in complete deactivation of the enzyme. These two residues potentially influenced enzyme activity by participating in shikimate binding.

Table S1. Enzymes used for the phylogenetic tree.

Enzyme	Species	ACCESSION	Enzyme	Species	ACCESSION
NtHCT	*Nicotiana tabacum*	Q8GSM7	NtHQT	*Nicotiana tabacum*	CAE46932.1
ArHCT1	*Actaea racemosa*	D4NUX0	NtTHT	*Nicotiana tabacum*	P80969
AsHHT1	*Avena sativa*	Q7XXP3	OsAHT1	*Oryza sativa* Japonica Group	Q7XPK7
AsHHT4	*Avena sativa*	A0A4Y5UJ70	OsHCT1	*Oryza sativa* subsp. japonica	Q0JBZ8
AtHCT	*Arabidopsis.thaliana*	Q9FI78	OsHCT2	*Oryza sativa* subsp. japonica	Q6K638
AtSHT	*Arabidopsis thaliana*	O64470	OsHCT4	*Oryza sativa* subsp. japonica	Q5SMM6
CcHCT	*Coffea canephora*	ABO47805.1	OsPHT1	*Oryza sativa* subsp. japonica	Q5SMM8
CcHQT	*Cynara cardunculus* var. *scolymus*	CAM84302.2	OsSHT1	*Oryza sativa* Japonica Group	Q2QRK9.1
CiHCT	*Cichorium intybus*	ANN12609.1	OsSHT2	*Oryza sativa* Japonica Group	Q0ING3.1
CkHCT	*Caragana korshinskii*	AHK05938.1	OsTBT1	*Oryza sativa* Japonica Group	Q2R0K3.1
CsHCT	*Coleus scutellarioides*	CBI83579.1	OsTHT1	*Oryza sativa* Japonica Group	Q338X7.1
CsRAS	*Coleus scutellarioides*	A0PDV5.1	PcHCT	*Phacelia campanularia*	QDF44405.1
DcHCBT 1	*Dianthus caryophyllus*	O24645	PcRAS	*Phacelia campanularia*	QDF44407.1
EpHCT	*Echinacea purpurea*	AXY97959.1	PvHCT2	*Panicum virgatum*	5FAL
EpHQT	*Echinacea purpurea*	QRI59127.1	SbHCT	*Sorghum bicolor*	4KEC_A
EsHCT	*Eleutherococcus senticosus*	UWM25202.1	SlHQT	*Solanum lycopersicum*	CAE46933.1
LaRAS	*Lavandula angustifolia*	AEA36976.1	SmHCT	*Salvia miltiorrhiza*	ACA64049.1
LjHQT	*Lonicera japonica*	ACZ52698.1	SmRAS	*Salvia miltiorrhiza*	ADA60182.1
MoRAS	*Melissa officinalis*	G0LD36	SnHCT	*Solanum nigrum*	QIC52996.1
NpHCT	*Narcissus pseudonarcissus*	A0A2H5AIZ1			

Table S2. PCR primers used for protein expression.

Primer	Sequence
pET-28a(+)-SUMO_*Ml*AT1-F	GAGAACAGATTGGTGGTACCGAGCTCATGAAAATCGAAGTGCGTG
ET-28a(+)-SUMO_*Ml*AT1-R	GTGGTGGTGGTGGTGGTGCTCGAGTTAGATTTCATACAGCAG
pET-28a(+)-SUMO_*Ml*AT4-F	CACAGAGAACAGATTGGTGGTACCGAGCTCATGTGCAGCATGAAAATTAG
pET-28a(+)-SUMO_*Ml*AT4-R	GTGGTGGTGGTGGTGGTGCTCGAGTTAAAAATCATACAGCAGT
pET-28a(+)_*Ml*AT6-F	CCGCGCGGCAGCCATATG
pET-28a(+)_*Ml*AT6-R	TTGTCGACGGAGCTCGAATTC
*Ml*AT6_R355A-F	GGTATTGTTGCAGCAGAAGGTCAT
*Ml*AT6_R355A-R	ATGACCTTCTGCTGCAACAATACC
*Ml*AT6_K398L-F	AGCGAAGGTCTGAGCTTTCTGATT
*Ml*AT6_K398L-R	AATCAGAAAGCTCAGACCTTCGCT

Information S1. The CDS sequences of *Ml*AT1-*Ml*AT7.

>*Ml*AT1

ATGAAGATCGAGGTGAGAGACTCGACGCTGGTGCGGCCGTCGGCGGCGACGCCGGCGGTCAGCC
TGTGGAATTCGAACGTTGACTTGGTGGTGCCTAACTTCCACACCCCGAGCGTCTACTTCTACCGCC
CCAACAGCGCCGCAGAGTTCTTCGACACGGCGGTGATGAAGGCGGCGCTCGGCCGCGCGCTGGT
GCCGTTCTACCCCATGGCGGGGAGGCTGAAGAGAGACGAAGACGGAAGGATCGAGATCGACTGCA
ACGCCGAGGGAGTGCTCTTTGTGGAGGCGGAGTCCGACGGCACGGTCGACGATTACGGCGATTTC
GCCCCCACTCTGGAGCTCCGTCGCCTCATTCCGGCGGTGGATTATTCGCAGGGAATCTCGGCCTA
CGCGCTTCTCGTGTTGCAGGTTACATTTTTCAAATGTGGTGGAGTATCCCTAGGCGTCGGTATGCAA
CACCACGTGGCTGACGGATTTTCCGGGCTCCACTTCATCAACGCGTGGTCCGACATGGCTCGTGG
GCTCGACATCACCCTCCCACCATTCATCGACCGGACCCTTCTCCGTGCCCGCGATCCCCCGCAGC
CGCAATTCAAGCATGTCGAGTACCAACCCCCTCCCTCCATGAAAACTTATGACGCAACGGAGACCG
TAGTCTCGATATTCAAGCTTACTCGCGAGCAACTCACCACTCTCAAAGCCAAGTCCAAGGACGACG
GCAATACGGTCAACTACAGCTCCTACGAGATGCTCTCGGGCCACGTCTGGCGCTGCGCCTGCCTG
GCCCGCGGCCTGCCCGAGGATCAGGACACGAAGCTGTACATCGCGACGGACGGGAGGTCCCGGC
TTCAGCCCCCGCTCCCGCCGGGCTACTTCGGGAACGTGATCTTCACGGCCACGCCCGTGGCCGTG
GCCGGGGACCTGGAGTCGAGGCCTGTCTGGTACGGCGCCAGCAAGATCCACGATGCGCTGGCCC
GGATGGACAATGAGTACCTGAGGTCGGCCCTCGACTACCTGGAGCTCCAGCCGGACCTCAAGGCG
CTTGTCCGCGGGGCCCACACGTTTAGGTGCCCGAACCTTGGGATCACGAGCTGGGTGCGGCTCCC
GATCCACGACGCGGATTTTGGGTGGGGGAGGCCCATCTTCATGGGGCCCGGGGGCATAGCGTAC
GAGGGTTTGAGCTTCGTGCTGCCGAGCCCGGCCAATGACGGAAGCCTGTCGGTTGCGATTTCCCT
TCAAGCAGAGCACATGAAGGTTTTTGAGAAGCTGCTCTATGAAATTTGA

>*Ml*AT2

ATGAAGATCAGTGCGAAAGAGTCGACGATGGTGAGGCCAACGACAGAGACGCCAGGTGGCAGTCT
GTGGCTGTCGAATTTGGACTTACTAATGCCGGCTTCCCACCACACCCGCACCGTCTACTTCTACGG
CTGCGAAGGTGCCGCCAACTTCTTCGACACAGCGGTGCTGAAGGAGGCGCTAGGGCGGGCCCTG
GTTGAGTTCTACCCTTATGCAGGGAGGCTGAGGAAGGCGGGCGACGGCCGCATCGAGATCGACTG
CAACGGAGAAGGAGTGTTGTTTGTGGAGGCGGAGTGCGATGGCGCCATCGACGATTTGGGCGGGT
TCGGACCCCGGTCGCCCGATCTCACTCTTGTGCCGAAAGTTGACTATTCTCAGGGAATTTCCACTTT
CCCACTTCTTTTGCTGCAGCTTACTCGCTTCAAATGCGGCGGCGTTTGCCTCGGCGTTGCAAATGA
GCATCACATTTCCGACGGGATCTCCGCCCTCCATTTCATCAACACATGGTCTGACCTCTCCCGCGG
CCTAACCACCGCGGTGCCCCCGTTCCTCGACCGCCGCCTCCTCTCCTCCCGCCACCCGCCGCACC
CGCAATTCCCCCACACCGAGCATCACCCGCCGCCGCCGCTGAAAACCCCTCTCCTGATCAGCTCCT
CTTACGCGACACAATCAACCTTCACCCTCACCCCCGACCACCTCAACCTCCTCAAACAAAAATGCGA
CCAAAACAAATCCGACCGCACGTACACCACGTACGAGGCGGTCGCCGGCCACGTGTGGCGCTGCG
CCACGATAGCCCGCAGCCTCCCCCACGACCAGGAAACCAAGCTCCAGATCCCGGTCGACGCGCGT
ACCCGCCTCCGGCCACCGCTTCCGCCGGGATTCTTCGGCAACGGGATCTTCTACACGTCGTCGAC
CGCGCTCTGCGGCGAGCTCGCGTCGAAACCCCTGGAATTCGCCGTCGGGAAAGTCAACGCGGCG
CTGGTCAAGATGGACGACGAGTACCTGAGGTCAGCGGTTGACTATCTGGAGCTGCAACTGCCGCG
TATCTACGAGATGGCGCGCAGCGAGAGGAACGTAAGGTGTCCGAATTTCGGGATAACGAGCTGGG
TCCGGCTGCCGTTCTACGAAGCGGATTTCGGGTGGGGAAAACCGGTTTATGCTGGACCGGCTGCG
GTTCAGTATGAAGGGAAGAGTTTGTTGCTGGTTGATAGGGAAAATGAAGGAGGCTTGGTGCTTGTG
ATCACGCTGCTTAAGCCGCATATGGAGGTTTTTGAGAAGTTGCTCTATGATATT

>*Ml*AT3

ATGAAGATCGAGGTGAGAGACTCGACGCTGGTGCGGCCGTCGGCGGCGACGCCGGCGGTCAGCC
TGTGGAATTCGAACGTTGACTTGGTGGTGCCTAACTTCCACACCCCGAGCGTCTACTTCTACCGCC
CCAACAGCGCCGCAGAGTTCTTCGACACGGCGGTGATGAAGGCGGCGCTCGGCCGCGCGCTGGT
GCCGTTCTACCCCATGGCGGGGAGGCTGAAGAGAGACGAAGACGGAAGGATCGAGATCGACTGCA
ACGCCGAGGGAGTGCTCTTTGTGGAGGCGGAGTCCGACGGCACGGTCGACGATTACGGCGATTTC
GCCCCCACTCTGGAGCTCCGTCGCCTCATTCCGGCGGTGGATTATTCGCAGGGAATCTCGGCCTA
CGCGCTTCTCGTGTTGCAGGTTACATTTTTCAAATGTGGTGGAGTATCCCTAGGCGTCGGTATGCAA
CACCACGTGGCTGACGGATTTTCCGGGCTCCACTTCATCAACGCGTGGTCCGACATGGCTCGTGG
GCTCGACATCACCCTCCCACCATTCATCGACCGGACCCTTCTCCGTGCCCGCGATCCCCCGCAGC
CGCAATTCAAGCATGTCGAGTACCAACCCCCTCCCTCCATGAAAACTTATGACGCAACGGAGACCG
TAGTCTCGATATTCAAGCTTACTCGCGAGCAACTCACCACTCTCAAAGCCAAGTCCAAGGACGACG
GCAATACGGTCAACTACAGCTCCTACGAGATGCTCTCGGGCCACGTCTGGCGCTGCGCCTGCCTG
GCCCGCGGCCTGCCCGAGGATCAGGACACGAAGCTGTACATCGCGACGGACGGGAGGTCCCGGC
TTCAGCCCCCGCTCCCGCCGGGCTACTTCGGGAACGTGATCTTCACGGCCACGCCCGTGGCCGTG
GCCGGGGACCTGGAGTCGAGGCCTGTCTGGTACGGCGCCAGCAAGATCCACGATGCGCTGGCCC
GGATGGACAATGAGTACCTGAGGTCGGCCCTCGACTACCTGGAGCTCCAGCCGGACCTCAAGGCG
CTTGTCCGCGGGGCCCACACGTTTAGGTGCCCGAACCTTGGGATCACGAGCTGGGTGCGGCTCCC
GATCCACGACGCGGATTTTGGGTGGGGGAGGCCCATCTTCATGGGGCCCGGGGGCATAGCGTAC
GAGGGTTTGAGCTTCGTGCTGCCGAGCCCGGCCAATGACGGAAGCCTGTCGGTTGCGATTTCCCT
TCAAGCAGAGCACATGAAGGTTTTTGAGAAGCTGCTCTATGAAATTTGA

>*Ml*AT4

ATGTGTAGCATGAAGATCAGTGTGAAGAAGACGACGGTGGTGAAGCCGATGGAAGAGACGCCGAG
CGGCGCTCTGTGGCTGTCGAGCTTGGACTTACTACTGCCTGCATCCTACCACTCCCGCACCGTCTA
CTTCTTCCGCTCCGATGGCTCCCCCGGCTTCTTCGACGCGGCGGCGCTCATGGCGGCGCTCAGCC
GGAATCTGGTTGAGTTCTACCCCTTTGCTGGGAGACTGAGGAGGGACGACAACGGCCGCCTCGAG
ATCAACTGCAACGGCGAAGGGGCGCTGTTCGTGGAGGCGGAGTGCGACGGCACGTTGGATGATAT
GGGTGATTTCAGCCCTAGGGCTGACGTCAAGTTCGTTCCCAAAGTGGACTATTCTCATGGAATCTC
CAGCTTCCCACTTTCCTTGATGCAGTTAACTCGCTTCAAGTGCGGTGGGGTCTGCTTAGGTGTTGTG
ACCGAGCACCACCTCGTAGATGGGATCGCCGCTCTCCACTACATCAACACGTGGGCCCGCATCGC
CCTCGGCGACAGGAACGCCGCCGCCGCGGACCCTCCGTTCTTGGACCGCCGCCTCCTCCTCTCCT
CCCGCCGGCCCCCGCAGCCAAAGTTCCCCCACACCGAGCACCAGCCTTTCCCTTCGCTCAAGGCC
CCACTAAACATCTCCGACGACACGTCATTCAAGATCCTCGCCCTCACCGGCAACCACCTCACCGCC
CTCAAGCAAGCATGCAACGGGAAGAAGGATCGGAGCTACACCACGTACGAGGCGGCGGCGGGCC
ACGTGTGGCGCTGCGTTTGCATGGCCCGCGGCCTGCCCTCCGATCAGGAGTCCAGGCTCCAGCTG
CCGGTTGACGGCCGGTCGCGGCTGCGGCCGCCGCTCCCGCCGCGTTACTTCGGCAACGGGATCT
TCTACACAACGTCGACCGTCACGTGCGGAGAGCTGGCCTCAAACCCGCTGGAGTACGCGGCCGGG
AAAGTGCGGGAGGCGGTGGCGCGGATGGACGACGAGTACTTGAGGTCGGCGGTGGACTACGTGG
AGATGAAGCTGCCGGAGTCCGACAGCATGGTGCGGAGTGATAAGAACGTGAAGTGTCCGAACTTC
GGGATAACGAGCTGGGTCCGGCTGCCGTTCTACGAGGCGGATTTCGGGTGGGGAAAACCGGTTTA
TGCCGGACCGGCTGCGGCTTTGTTCGAAGGGAAAAGCTTTTGGTTTGTTGATCCTAAAAAGGAAGG
AGACACATTGCTTGCGATCACGTTGCTTAAACCGCATATGGAGCTATTTGAGAAGTTACTCTATGATT
TTTGA

>*Ml*AT5

AACACATAACACGTACTACTACTCATTCCTACTATTCATCACCAAGGACCTTCGACGATGAAGATTAC
GGTGAAGGAGTCGGCGGTGGTGAAGCCAACTCCGGCGAAGCCGCGGACGACCATATGGATCTCC
AATCTCGACGCCATTTTGCCGGAGAACTACCACACTCGCACCCACTACTTCTACCGCCCAACCGCC
GCCGCCGACTTCTTCGACGCGGCGGTGCTCACGTCGGCGCTCGCTCGGACTCTCTCCGAGTTCTA
CCCCGTGGCCGGGAGGCTGAAGAAGGACGGTAACGGCCGCGTAGAGATCGACTGCAGCGGCGAG
GGGGCGGTGTTCGTGGAGGCGGAGGCCGACGGCGAGATCGACGATTTAGGGGATTTCTCCCCAA
ACCCTAACATCTGCCTCGCCCCCAAAGTCGACTATTCTCAAGGAATTTCATCTTTCCCGCTGTTATTA
GTTCAGGTTACCAGATTCAAATGCGGTGGAGTTTGCTTGGGCGTTGCGATGGACCATCAGGTGAAA
GACGGAATTTCCGCTCTGCATATTATCCACACATGGTGCGATATAGCCCGCGGCCTCGACATCGCC
GTGCCGCCGTACATGGACCGCCGTGTCCTGGCGGCGCGTGAACCGCCGCAGCCTAAGTTCGAGC
ACGTCGAATTCCATCCTCCGCCGCCGCTGAAAAACTCAGAAGCTCACACAAATGGTTCGGAAACAA
AATTTGCAGTACTGAAGCTAACGCGGGAGCAGCTAATTATCCTCAAAGGCAACAGCCAAGAAGATG
ATGGAAAGAGGTCTCCTTACAGTTCATTCGAAGCGCTCACAGGGCATGTTTGGAGGTGCATCTGCA
AAGCTCGTCGGCTACCGGAAGATCAAGAAACAAAGCTGACTATTATAGTGGACGGCCGATCGAGGC
TGCGACCCCCGCTGCCACCCGGATACTTCGGCAATGCGGTGTTCAAAGCCACACACATCGCTTTGA
GCCGTGAAGTCGAATCGAACCCGTTGAAATATGTGGTTTGCAAAGTTCGCGAGGCCTTGACCCGGA
TGGACGACGAGTACTTGAGGTCCGCAATCGATTACTTGGAGGTCGAGGGCGGACTCGGCCCAAAT
GCACGAGGCACCGGGCTTTACAAAAGCCCGAACCTCGGGATCACTAGTTGGGCCCGACTGCCCTT
CTACGAAGCGGATTTCGGATGGGGTAGGCCGTTTCACGTGGGGCTCGGCGCCGTTCCAGCCGAAG
GGCATCTGATCGTGATGCCTAGTCCGACGAACGACGGCGGCTTGCGCCTGGCGATCGCGCTGCCC
GAGGAGCAGATGAAGATGTTCGAGAAGATCTTCTACGAAATATGATGTTTCCCGAGCAAGTGTTGCA
CTAGTTAATTAAAGTTGATTTGCCAACTTTCGTATTATTAGATTAGATGTTTGCATTTTTGAATATATA
GAACGTGGCGAGTATTATTAATAAAGTAAAACAAATGTTCACGTGCTAGTTGTGGGACAGTTTGCTT
GATTTGTGAGACTTTTAATTCAAACTAGTTTTGGTGCCCCGGCTTCGCTCGGGTAATACTATCAGCT
CTTTTTTCTTCTTTGAATAATTTAATTTGTACATTAGTTGGTGTGTTCTTTATAATTTTGTAAAATTAATA
AGTTTTTCATA

>*Ml*AT6

ATGAAGATCTTAATGAAGGAGTCCACGATGGTAAAGCCGATGGAGAAAACGCCGAGCGGCAGTGT
GTGGCTCTCCAGTTTAGACTTAGTTTTTCCGGCCACCTACCATACTCGCTGGGTAAACTTCTACCGT
TCCGACAGCGCCGCCAACTTTTTCGATACGGCGATGCTGAAGGCGGCGCTTAGCCGCACCTTGGT
TGAGTTCTACCCTTATGCCGGGAGGCTGAGAAAGGACGATAACGGCCGCATCGAGATCAACTGCA
GCAACGAAGGGGTACTATTCATAGAGGCGGAGTGCAACGGCGCCGTCAACGACTTGGGTGGTTTC
AGCCCTATGCCCGACATCAGCCTCGTACCCATAGTCGATTATTCTAACGGAATTTCATCCTTCCCAC
TTTTTCTAATACAGTTAACTCGGTTCAAATGCGGTGGAGTGAGCTTAGGTTTCGCAAACGAGCATCA
CGTGTCAGATGGGGTCTCTGTTCTCCATTTCGTCATCACGTGGTCGGAAATCGCTCGTGGCCTAAC
TCCCGCCGTCCCTCCCTCCTTGGACCGCCGCCTCCTCTTCCCCCGAAACCCTCCGCGGCCGCAGT
TCCCCCACATCGAGCACCGCCCCCCTCCAACACTCAAAACCCCTCTCGCCAACTCAGACACGTCAT
TCTCATCATTCAGACTCACCCCCGTCCACATCAACGCCCTCAAACAAAAATGCAGACGCAACAGCG
ACAGCGACAGTAGTCCGAGCTTTACCGGGTACGAGGTTGTCGCCGGCCACGTGTGGCGCTGCATC
AGCATCGCCCGCGGGCTCCCGGTAGATCAGGAGACCAAGCTGCAGCTCCCGGTCGACTCCCGGC
GGAAGCTACGGCCGCCGCTCCCGCCGGGGTTCTTCGGCAATGGGATCTTCTACACCGGCGCGTTC
ACTTCCTGCGGCGAGCTGGCGTCTAAGCCGCTGAGGTTCGCGGTGGGGAAAGTGCGCGAGGCGA

TTGCCCGGATGGACGACGAGTACTTGCGCTCGGCGGTGGATTACGTGGAGTTGAATATGGCGAAG
GTGGAGGGCATCGTGCGCGCCGAAGGGCACGTGAGATGCCCCAACTTCGGGATCACGAGCTGGG
TTCGGATGCCTGCGTACGAAGTGGACTTCGGGTGGGGGAAGCCGGTTTATGTGGGGCCAGGGGC
AATTATGTCCGAAGGGAAAAGTTTTCTGATCGCTGATCCTCAAAATGAGGGGGGCGTGCTGCTGCT
TATCACGCTCCATAAACAGCACATGAAGGAATTTGAGAAGCTTCTCTATGCTGAGTTGCTCTTCCTC
AAGAGTAATATT

>*Ml*AT7

ATGAAGATCGATGTGAAAGAGTCGACGATGATAAAGCCGGCGGCGGAGACGCCGAGCGACAGCAT
ATGGCTGTCGAATCTGGACCTGCTATCGCCGGCGAACTACCACACACTGAGCGTCCACTTCTACCG
CCACGACGGCTCAGCCAACTTCTTCGACGCGGCGACGTTGAAGGAAGCTCTCGGCCGCGCGCTGG
TGGAGTTCTACCCGTACGCCGGTCGGCTGAAGCCGAACGGCAACCGCCTAGAGATCAACTGCAAC
GGCGAGGGGCTGCTGCTGGTGGAGGCGGAGTGCGACGGCGCCTTGGATGAGCTGGGGGACTTC
GCCCCCCGCCCCGATCTCAACCTTATCCCCAAAATTGATTATGCCAAGGGTATTTCCACGTACCCTC
TCATCGTTTTTCAGGTAAATAAATCTATTTCTACTCCTTTAATTTACTTTCCCTTCAAAGAAAGTTTTTA
CTTTATTTTTTTTATTATTTAA

Information S2. The optimized sequences of *Ml*AT1, *Ml*AT2, *Ml*AT4 and *Ml*AT6.

>*Ml*AT1

ATGAAAATCGAAGTGCGTGATAGCACACTGGTTCGTCCGAGCGCAGCAACACCTGCAGTTAGTCTG
TGGAACAGCAATGTTGATCTGGTTGTGCCGAATTTTCATACCCCGAGCGTTTATTTTTATCGTCCTAA
TAGCGCAGCAGAATTTTTTGATACCGCAGTTATGAAAGCAGCACTGGGTCGTGCGCTGGTGCCTTT
TTATCCGATGGCAGGCCGTCTGAAACGTGATGAAGATGGTCGCATTGAAATCGATTGTAATGCAGA
AGGTGTGCTGTTTGTTGAAGCCGAAAGTGATGGTACCGTTGATGATTATGGTGATTTTGCACCGACT
CTGGAACTGCGTCGTCTGATTCCGGCCGTTGATTATAGCCAGGGTATTAGTGCATATGCCCTGCTG
GTGCTGCAGGTTACGTTTTTTAAATGTGGTGGTGTTAGCCTGGGTGTTGGTATGCAGCATCATGTTG
CCGATGGTTTTAGTGGTCTGCATTTTATTAACGCGTGGAGCGATATGGCGCGTGGTCTGGATATTAC
GCTGCCGCCTTTTATTGATCGTACTCTGCTGCGTGCCCGTGATCCTCCTCAGCCTCAGTTTAAACAT
GTTGAATATCAGCCGCCGCCGTCAATGAAAACCTATGATGCTACCGAAACCGTTGTTTCCATTTTTA
AACTGACCCGTGAACAGCTGACTACCCTGAAAGCAAAAAGCAAAGATGATGGTAATACCGTTAATTA
TAGCAGCTATGAAATGCTGAGCGGTCATGTTTGGCGTTGTGCCTGTCTGGCACGTGGTCTGCCTGA
AGATCAGGATACCAAACTGTATATTGCGACGGATGGTCGTAGCCGTCTGCAGCCTCCTCTGCCTCC
TGGTTATTTTGGTAATGTGATTTTTACAGCCACCCCGGTTGCAGTTGCAGGTGATCTGGAAAGCCGT
CCGGTTTGGTATGGTGCTTCTAAAATTCATGATGCACTGGCACGTATGGATAATGAATATCTGCGCT
CTGCCCTGGATTATCTGGAACTGCAGCCGGATCTGAAAGCACTGGTTCGTGGTGCGCATACCTTTC
GTTGCCCGAACCTGGGTATTACTTCATGGGTTCGTCTGCCGATTCATGATGCCGATTTTGGTTGGG
GTCGTCCGATTTTTATGGGTCCGGGTGGTATTGCATATGAAGGTCTGTCTTTTGTTCTGCCGTCTCC
GGCGAATGATGGTAGTCTGTCTGTTGCTATTAGTCTGCAGGCGGAACATATGAAAGTTTTTGAAAAA
CTGCTGTATGAAATCTAA

>*Ml*AT2

ATGAAAATCAGCGCAAAAGAAAGCACCATGGTCCGTCCAACCACAGAAACACCGGGTGGTAGCCTG
TGGCTGAGTAACCTGGATCTGCTGATGCCGGCAAGCCATCATACCCGTACCGTTTATTTTTATGGTT
GCGAAGGTGCAGCGAATTTTTTTGATACCGCAGTCCTGAAAGAAGCACTGGGTCGTGCACTGGTTG
AATTTTATCCGTATGCAGGTCGCCTGCGTAAAGCAGGTGATGGTCGTATTGAAATTGATTGTAATGG

TGAAGGTGTGCTGTTTGTTGAAGCGGAATGTGATGGTGCAATTGATGATCTGGGCGGCTTTGGTCC
GCGTAGTCCTGATCTGACACTGGTTCCGAAAGTTGATTATAGCCAGGGTATTAGCACGTTTCCTCTG
CTGCTGCTGCAGCTGACGCGTTTTAAATGTGGTGGTGTTTGTCTGGGTGTTGCAAATGAACATCATA
TTAGCGATGGTATTAGTGCACTGCATTTTATTAACACATGGTCTGATCTGTCTCGTGGTCTGACCAC
CGCAGTGCCTCCTTTTCTGGATCGTCGTCTGCTGAGTTCCCGTCATCCGCCTCATCCTCAGTTTCCG
CATACCGAACATCATCCTCCACCGCCTCTGAAAACCCCTCTGCTGATTTCAAGTTCTTATGCAACCC
AGAGCACCTTTACCCTGACCCCTGATCATCTGAATCTGCTGAAACAGAAATGTGATCAGAACAAATC
CGATCGTACCTATACCACCTATGAAGCAGTTGCCGGCCATGTTTGGCGCTGTGCAACAATTGCCCG
TAGCCTGCCTCATGATCAGGAAACAAAACTGCAGATCCCGGTAGATGCACGTACCCGTCTGCGTCC
TCCACTGCCTCCTGGTTTTTTTGGTAATGGTATTTTTTATACAAGTAGTACCGCACTGTGTGGTGAAC
TGGCAAGCAAACCTCTGGAATTTGCCGTTGGTAAAGTTAATGCAGCACTGGTTAAAATGGATGATGA
ATATCTGCGTAGTGCAGTGGATTATCTGGAACTGCAGCTGCCGCGTATTTATGAAATGGCACGTTCT
GAACGTAATGTTCGTTGTCCGAATTTTGGTATTACCAGCTGGGTTCGTCTGCCGTTTTATGAAGCAG
ATTTTGGTTGGGGTAAACCGGTTTATGCTGGTCCGGCGGCAGTTCAGTATGAAGGTAAAAGTCTGC
TGCTGGTTGATCGTGAAAACGAAGGTGGTCTGGTTCTGGTTATTACCCTGCTGAAACCGCATATGG
AAGTTTTTGAAAAACTGCTGTATGATATCTAA

>*Ml*AT4

ATGTGCAGCATGAAAATTAGCGTTAAAAAGACCACCGTTGTTAAACCGATGGAAGAAACACCGAGC
GGCGCACTGTGGCTGAGTAGTCTGGATCTGCTGCTGCCGGCAAGCTATCATAGCCGTACCGTCTAT
TTTTTTCGTAGCGATGGCAGCCCTGGTTTTTTTGATGCAGCAGCCCTGATGGCAGCACTGAGTCGTA
ATCTGGTTGAATTTTATCCGTTTGCAGGTCGTCTGCGTCGTGATGATAATGGTCGTCTGGAAATTAA
TTGTAATGGTGAAGGTGCGCTGTTTGTTGAAGCAGAATGTGATGGTACCCTGGATGATATGGGTGA
TTTTAGCCCGCGTGCAGATGTTAAATTTGTTCCGAAAGTTGATTATAGCCATGGTATTAGTAGCTTTC
CGCTGTCTCTGATGCAGCTGACGCGTTTTAAATGTGGTGGTGTTTGTCTGGGTGTTGTTACTGAACA
TCATCTGGTTGATGGTATTGCGGCACTGCATTATATTAATACCTGGGCCCGTATTGCCCTGGGTGAT
CGTAATGCTGCAGCCGCAGATCCGCCTTTTCTGGATCGTCGTCTGCTGCTGAGTAGTCGTCGTCCT
CCTCAGCCTAAATTTCCGCATACAGAACATCAGCCTTTTCCGTCACTGAAAGCACCGCTGAATATTT
CTGATGATACCTCTTTTAAAATCCTGGCCCTGACCGGTAATCATCTGACCGCACTGAAACAGGCCTG
TAACGGCAAAAAGGATCGTAGTTATACCACCTATGAAGCAGCAGCAGGCCATGTTTGGCGCTGTGT
TTGCATGGCTCGTGGTCTGCCTAGCGATCAGGAAAGCCGTCTGCAGCTGCCTGTGGATGGTCGTA
GTCGTCTGCGTCCTCCTCTGCCTCCTCGTTATTTTGGTAATGGTATTTTTTATACAACAAGTACCGTG
ACCTGTGGTGAACTGGCGAGCAATCCTCTGGAATATGCGGCAGGTAAAGTTCGTGAAGCAGTTGCA
CGTATGGATGATGAATATCTGCGTAGTGCCGTTGATTATGTTGAAATGAAACTGCCGGAAAGCGATA
GTATGGTGCGTAGCGATAAAAATGTTAAATGCCCGAATTTTGGTATCACAAGCTGGGTGCGTCTGCC
GTTTTATGAAGCAGATTTTGGTTGGGGTAAACCGGTTTATGCAGGTCCGGCGGCAGCTCTGTTTGA
AGGTAAAAGTTTTTGGTTTGTTGATCCGAAAAAGGAAGGTGATACACTGCTGGCCATTACCCTGCTG
AAACCGCATATGGAACTGTTTGAAAAACTGCTGTATGATTTTTAA

>*Ml*AT6

ATGAAAATCCTGATGAAAGAAAGCACCATGGTTAAACCGATGGAAAAAACCCCGAGCGGTAGCGTT
TGGCTGAGCAGCCTGGATCTGGTTTTTCCGGCAACCTATCATACCCGTTGGGTTAATTTTTATCGTA
GCGATAGCGCAGCAAATTTTTTTGATACCGCAATGCTGAAAGCAGCACTGAGCCGTACCCTGGTTG
AATTTTATCCGTATGCAGGTCGTCTGCGTAAAGATGATAATGGTCGTATTGAAATTAATTGTAGCAAT
GAAGGTGTTCTGTTTATTGAAGCAGAATGTAATGGTGCAGTTAATGATCTGGGTGGTTTTAGCCCGA

TGCCGGATATTAGCCTGGTTCCGATTGTTGATTATAGCAATGGTATTAGCAGCTTTCCGCTGTTTCT
GATTCAGCTGACCCGTTTTAAATGTGGTGGTGTTAGCCTGGGTTTTGCAAATGAACATCATGTTAGC
GATGGTGTTAGCGTTCTGCATTTTGTTATTACCTGGAGCGAAATTGCACGTGGTCTGACCCCGGCA
GTTCCGCCGAGCCTGGATCGTCGTCTGCTGTTTCCGCGTAATCCGCCGCGTCCGCAGTTTCCGCAT
ATTGAACATCGTCCGCCGCCGACCCTGAAAACCCCGCTGGCAAATAGCGATACCAGCTTTAGCAGC
TTTCGTCTGACCCCGGTTCATATTAATGCACTGAAACAGAAATGTCGTCGTAATAGCGATAGCGATA
GCAGCCCGAGCTTTACCGGTTATGAAGTTGTTGCAGGTCATGTTTGGCGTTGTATTAGCATTGCACG
TGGTCTGCCGGTTGATCAGGAAACCAAACTGCAGCTGCCGGTTGATAGCCGTCGTAAACTGCGTCC
GCCGCTGCCGCCGGGTTTTTTTGGTAATGGTATTTTTTATACCGGTGCATTTACCAGCTGTGGTGAA
CTGGCAAGCAAACCGCTGCGTTTTGCAGTTGGTAAAGTTCGTGAAGCAATTGCACGTATGGATGAT
GAATATCTGCGTAGCGCAGTTGATTATGTTGAACTGAATATGGCAAAAGTTGAAGGTATTGTTCGTG
CAGAAGGTCATGTTCGTTGTCCGAATTTTGGTATTACCAGCTGGGTTCGTATGCCGGCATATGAAGT
TGATTTTGGTTGGGGTAAACCGGTTTATGTTGGTCCGGGTGCAATTATGAGCGAAGGTAAAAGCTTT
CTGATTGCAGATCCGCAGAATGAAGGTGGTGTTCTGCTGCTGATTACCCTGCATAAACAGCATATGA
AAGAATTTGAAAAACTGCTGTATGCAGAACTGCTGTTTCTGAAAAGCAATATT

Information S3. The nucleotide sequences of *Ml*AT6 mutants.

>*Ml*AT6 R355A

ATGAAAATCCTGATGAAAGAAAGCACCATGGTTAAACCGATGGAAAAAACCCCGAGCGGTAGCGTT
TGGCTGAGCAGCCTGGATCTGGTTTTTCCGGCAACCTATCATACCCGTTGGGTTAATTTTTATCGTA
GCGATAGCGCAGCAAATTTTTTTGATACCGCAATGCTGAAAGCAGCACTGAGCCGTACCCTGGTTG
AATTTTATCCGTATGCAGGTCGTCTGCGTAAAGATGATAATGGTCGTATTGAAATTAATTGTAGCAAT
GAAGGTGTTCTGTTTATTGAAGCAGAATGTAATGGTGCAGTTAATGATCTGGGTGGTTTTAGCCCGA
TGCCGGATATTAGCCTGGTTCCGATTGTTGATTATAGCAATGGTATTAGCAGCTTTCCGCTGTTTCT
GATTCAGCTGACCCGTTTTAAATGTGGTGGTGTTAGCCTGGGTTTTGCAAATGAACATCATGTTAGC
GATGGTGTTAGCGTTCTGCATTTTGTTATTACCTGGAGCGAAATTGCACGTGGTCTGACCCCGGCA
GTTCCGCCGAGCCTGGATCGTCGTCTGCTGTTTCCGCGTAATCCGCCGCGTCCGCAGTTTCCGCAT
ATTGAACATCGTCCGCCGCCGACCCTGAAAACCCCGCTGGCAAATAGCGATACCAGCTTTAGCAGC
TTTCGTCTGACCCCGGTTCATATTAATGCACTGAAACAGAAATGTCGTCGTAATAGCGATAGCGATA
GCAGCCCGAGCTTTACCGGTTATGAAGTTGTTGCAGGTCATGTTTGGCGTTGTATTAGCATTGCACG
TGGTCTGCCGGTTGATCAGGAAACCAAACTGCAGCTGCCGGTTGATAGCCGTCGTAAACTGCGTCC
GCCGCTGCCGCCGGGTTTTTTTGGTAATGGTATTTTTTATACCGGTGCATTTACCAGCTGTGGTGAA
CTGGCAAGCAAACCGCTGCGTTTTGCAGTTGGTAAAGTTCGTGAAGCAATTGCACGTATGGATGAT
GAATATCTGCGTAGCGCAGTTGATTATGTTGAACTGAATATGGCAAAAGTTGAAGGTATTGTTGCAG
CAGAAGGTCATGTTCGTTGTCCGAATTTTGGTATTACCAGCTGGGTTCGTATGCCGGCATATGAAGT
TGATTTTGGTTGGGGTAAACCGGTTTATGTTGGTCCGGGTGCAATTATGAGCGAAGGTAAAAGCTTT
CTGATTGCAGATCCGCAGAATGAAGGTGGTGTTCTGCTGCTGATTACCCTGCATAAACAGCATATGA
AAGAATTTGAAAAACTGCTGTATGCAGAACTGCTGTTTCTGAAAAGCAATATTTAA

>*Ml*AT6 K398L

ATGAAAATCCTGATGAAAGAAAGCACCATGGTTAAACCGATGGAAAAAACCCCGAGCGGTAGCGTT
TGGCTGAGCAGCCTGGATCTGGTTTTTCCGGCAACCTATCATACCCGTTGGGTTAATTTTTATCGTA
GCGATAGCGCAGCAAATTTTTTTGATACCGCAATGCTGAAAGCAGCACTGAGCCGTACCCTGGTTG
AATTTTATCCGTATGCAGGTCGTCTGCGTAAAGATGATAATGGTCGTATTGAAATTAATTGTAGCAAT

GAAGGTGTTCTGTTTATTGAAGCAGAATGTAATGGTGCAGTTAATGATCTGGGTGGTTTTAGCCCGA
TGCCGGATATTAGCCTGGTTCCGATTGTTGATTATAGCAATGGTATTAGCAGCTTTCCGCTGTTTCT
GATTCAGCTGACCCGTTTTAAATGTGGTGGTGTTAGCCTGGGTTTTGCAAATGAACATCATGTTAGC
GATGGTGTTAGCGTTCTGCATTTTGTTATTACCTGGAGCGAAATTGCACGTGGTCTGACCCCGGCA
GTTCCGCCGAGCCTGGATCGTCGTCTGCTGTTTCCGCGTAATCCGCCGCGTCCGCAGTTTCCGCAT
ATTGAACATCGTCCGCCGCCGACCCTGAAAACCCCGCTGGCAAATAGCGATACCAGCTTTAGCAGC
TTTCGTCTGACCCCGGTTCATATTAATGCACTGAAACAGAAATGTCGTCGTAATAGCGATAGCGATA
GCAGCCCGAGCTTTACCGGTTATGAAGTTGTTGCAGGTCATGTTTGGCGTTGTATTAGCATTGCACG
TGGTCTGCCGGTTGATCAGGAAACCAAACTGCAGCTGCCGGTTGATAGCCGTCGTAAACTGCGTCC
GCCGCTGCCGCCGGGTTTTTTTGGTAATGGTATTTTTTATACCGGTGCATTTACCAGCTGTGGTGAA
CTGGCAAGCAAACCGCTGCGTTTTGCAGTTGGTAAAGTTCGTGAAGCAATTGCACGTATGGATGAT
GAATATCTGCGTAGCGCAGTTGATTATGTTGAACTGAATATGGCAAAAGTTGAAGGTATTGTTCGTG
CAGAAGGTCATGTTCGTTGTCCGAATTTTGGTATTACCAGCTGGGTTCGTATGCCGGCATATGAAGT
TGATTTTGGTTGGGGTAAACCGGTTTATGTTGGTCCGGGTGCAATTATGAGCGAAGGTCGTAGCTTT
CTGATTGCAGATCCGCAGAATGAAGGTGGTGTTCTGCTGCTGATTACCCTGCATAAACAGCATATGA
AAGAATTTGAAAAACTGCTGTATGCAGAACTGCTGTTTCTGAAAAGCAATATTTAA

>*Ml*AT6 R355A+K398L

ATGAAAATCCTGATGAAAGAAAGCACCATGGTTAAACCGATGGAAAAAACCCCGAGCGGTAGCGTT
TGGCTGAGCAGCCTGGATCTGGTTTTTCCGGCAACCTATCATACCCGTTGGGTTAATTTTTATCGTA
GCGATAGCGCAGCAAATTTTTTTGATACCGCAATGCTGAAAGCAGCACTGAGCCGTACCCTGGTTG
AATTTTATCCGTATGCAGGTCGTCTGCGTAAAGATGATAATGGTCGTATTGAAATTAATTGTAGCAAT
GAAGGTGTTCTGTTTATTGAAGCAGAATGTAATGGTGCAGTTAATGATCTGGGTGGTTTTAGCCCGA
TGCCGGATATTAGCCTGGTTCCGATTGTTGATTATAGCAATGGTATTAGCAGCTTTCCGCTGTTTCT
GATTCAGCTGACCCGTTTTAAATGTGGTGGTGTTAGCCTGGGTTTTGCAAATGAACATCATGTTAGC
GATGGTGTTAGCGTTCTGCATTTTGTTATTACCTGGAGCGAAATTGCACGTGGTCTGACCCCGGCA
GTTCCGCCGAGCCTGGATCGTCGTCTGCTGTTTCCGCGTAATCCGCCGCGTCCGCAGTTTCCGCAT
ATTGAACATCGTCCGCCGCCGACCCTGAAAACCCCGCTGGCAAATAGCGATACCAGCTTTAGCAGC
TTTCGTCTGACCCCGGTTCATATTAATGCACTGAAACAGAAATGTCGTCGTAATAGCGATAGCGATA
GCAGCCCGAGCTTTACCGGTTATGAAGTTGTTGCAGGTCATGTTTGGCGTTGTATTAGCATTGCACG
TGGTCTGCCGGTTGATCAGGAAACCAAACTGCAGCTGCCGGTTGATAGCCGTCGTAAACTGCGTCC
GCCGCTGCCGCCGGGTTTTTTTGGTAATGGTATTTTTTATACCGGTGCATTTACCAGCTGTGGTGAA
CTGGCAAGCAAACCGCTGCGTTTTGCAGTTGGTAAAGTTCGTGAAGCAATTGCACGTATGGATGAT
GAATATCTGCGTAGCGCAGTTGATTATGTTGAACTGAATATGGCAAAAGTTGAAGGTATTGTTGCAG
CAGAAGGTCATGTTCGTTGTCCGAATTTTGGTATTACCAGCTGGGTTCGTATGCCGGCATATGAAGT
TGATTTTGGTTGGGGTAAACCGGTTTATGTTGGTCCGGGTGCAATTATGAGCGAAGGTCGTAGCTTT
CTGATTGCAGATCCGCAGAATGAAGGTGGTGTTCTGCTGCTGATTACCCTGCATAAACAGCATATGA
AAGAATTTGAAAAACTGCTGTATGCAGAACTGCTGTTTCTGAAAAGCAATATTTAA
